# Stabilization of TGF‐β Receptor 1 by a Receptor‐Associated Adaptor Dictates Feedback Activation of the TGF‐β Signaling Pathway to Maintain Liver Cancer Stemness and Drug Resistance

**DOI:** 10.1002/advs.202402327

**Published:** 2024-07-09

**Authors:** Kewei Liu, Fanxuan Tian, Xu Chen, Biyin Liu, Shuoran Tian, Yongying Hou, Lei Wang, Mengyi Han, Shiying Peng, Yuting Tan, Yuwei Pan, Zhaole Chu, Jinyang Li, Linrong Che, Dongfeng Chen, Liangzhi Wen, Zhongyi Qin, Xianfeng Li, Junyu Xiang, Xiu‐wu Bian, Qin Liu, Xiaoli Ye, Tao Wang, Bin Wang

**Affiliations:** ^1^ Engineering Research Center of Coptis Development and Utilization (Ministry of Education), School of Life Sciences Southwest University Chongqing 400715 P. R. China; ^2^ Department of Gastroenterology, Chongqing Key Laboratory of Digestive Malignancies, Daping Hospital Army Medical University (Third Military Medical University) Chongqing 400042 P. R. China; ^3^ School of Medicine Chongqing University Chongqing 400044 P. R. China; ^4^ Department of Pathology Daping Hospital, Army Medical University (Third Military Medical University) Chongqing 400042 P. R. China; ^5^ Institute of Pathology and Southwest Cancer Center, and Key Laboratory of Tumor Immunopathology of Ministry of Education of China, Southwest Hospital Army Medical University (Third Military Medical University) Chongqing 400038 P. R. China; ^6^ Jinfeng Laboratory Chongqing 401329 P. R. China

**Keywords:** cancer stem cells, feedback regulation, TGF‐β receptor, TGF‐β signaling, tyrosine kinase inhibitor

## Abstract

Dysregulation of the transforming growth factor‐β (TGF‐β) signaling pathway regulates cancer stem cells (CSCs) and drug sensitivity, whereas it remains largely unknown how feedback regulatory mechanisms are hijacked to fuel drug‐resistant CSCs. Through a genome‐wide CRISPR activation screen utilizing stem‐like drug‐resistant properties as a readout, the TGF‐β receptor‐associated binding protein 1 (TGFBRAP1) is identified as a TGF‐β‐inducible positive feedback regulator that governs sensitivity to tyrosine kinase inhibitors (TKIs) and promotes liver cancer stemness. By interacting with and stabilizing the TGF‐β receptor type 1 (TGFBR1), TGFBRAP1 plays an important role in potentiating TGF‐β signaling. Mechanistically, TGFBRAP1 competes with E3 ubiquitin ligases Smurf1/2 for binding to TGFΒR1, leading to impaired receptor poly‐ubiquitination and proteasomal degradation. Moreover, hyperactive TGF‐β signaling in turn up‐regulates TGFBRAP1 expression in drug‐resistant CSC‐like cells, thereby constituting a previously uncharacterized feedback mechanism to amplify TGF‐β signaling. As such, TGFBRAP1 expression is correlated with TGFΒR1 levels and TGF‐β signaling activity in hepatocellular carcinoma (HCC) tissues, as well as overall survival and disease recurrence in multiple HCC cohorts. Therapeutically, blocking TGFBRAP1‐mediated stabilization of TGFBR1 by selective inhibitors alleviates Regorafenib resistance via reducing CSCs. Collectively, targeting feedback machinery of TGF‐β signaling pathway may be an actionable approach to mitigate drug resistance and liver cancer stemness.

## Graphical abstract

TGFBRAP1 protects TGFBR1 from ubiquitination and degradation by competing with SMURF1/2 for binding to TGFBR1, thereby promoting the activation of the TGF‐β signaling pathway and enhancing cancer stemness and resistance to regorafenib in HCC. Moreover, TGFBRAP1 is transcriptionally up‐regulated by the SMAD2/3 complex, thus forming a positive feedback regulation of the TGF‐β signaling pathway.

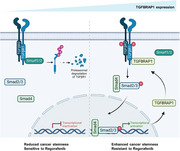

## 1 Introduction

The transforming growth factor‐β (TGF‐β) signaling pathway is essential for embryonic development, tissue homeostasis, and regeneration through coordinating cell fate commitment and functional plasticity.^[^ ^1^ ^]^ Aberrancies of TGF‐β signaling, particularly in various epithelial tissues, contribute to human tumorigenesis and therapeutic resistance by acting on a subpopulation of cancer stem cells (CSCs).^[^ ^2^, ^3^, ^4^ Active TGF‐β1 binds to TGF‐β receptor type 2 (R2, TGFBR2), which forms a heteromeric complex with TGF‐β receptor type I (R1, TGFBR1) to mediates its phosphorylation. The ligand‐activated receptor complex phosphorylates and activates SMAD2 and SMAD3, which together form a complex that subsequently translocate into the nucleus and acts as key cofactors in the transcriptional activation of target genes.^[^ ^1^, ^5^ ^]^ As such, soluble TGF‐β1, either produced in an autocrine fashion or released from the tumor microenvironment, plays important roles in dictating the stem‐like drug‐resistant properties in a variety of human cancers.^[^ ^2^, ^6^ ^]^ For example, TGF‐β induces the expression of SOX2 and enhances cancer stemness and drug resistance in hepatocellular carcinoma (HCC) and oral squamous cell carcinoma.^[^ ^4^, ^7^ ^]^ Hyperactivation of TGF‐β signaling also promotes resistance to anti‐cancer drugs in multiple caner types including colorectal cancer (CRC).^[^ ^8^ ^]^ Targeting TGF‐β signaling in advanced cancers thus represents an opportunity to eliminate CSCs and enhance therapeutic efficacy.

Feedback control of TGF‐β signaling offers an important layer of regulation to maintain homeostasis of the signaling pathway, while defects of feedback machinery by various oncogenic events leads to its hyperactivation in human cancers. Inhibitory SMADs, including SMAD6 and SMAD7, form a negative feedback loop to restrain the activity of the TGF‐β signaling pathway. However, epigenetic silencing of *SMAD6* and *SMAD7* hyperactivates TGF‐β signaling pathway and promotes cancer stemness and drug resistance in multiple malignancies.^[^ ^9^, ^10^, ^11^ ^]^ The TGF‐β‐induced LncRNA UTGF stabilizes *SMAD2* and *SMAD4* transcripts to enhance stem cell self‐renewal in a positive feedback manner.^[^ ^12^ ^]^ Induction of periostin (POSTN), an extracellular matrix (ECM)‐associated multi‐functional protein, by TGF‐β signaling leads to the release of TGF‐β from the ECM to amplify downstream signaling in a positive feedback loop, thus enhancing stemness and drug resistance of HCC.^[^ ^13^ ^]^ While the TGF‐β signaling pathway is often activated in advanced cancers, it is still poorly understood how feedback regulatory mechanisms are exploited in CSCs to confer therapeutic resistance.

HCC, the major type of primary liver cancers, is highly aggressive with dismal clinical outcomes. More than half of HCC patients are diagnosed at advanced stages and often incurable by surgery, where molecularly targeted therapy, via tyrosine kinase inhibitors, represents an important therapeutic modality. In this regard, Regorafenib is a widely used in treating advanced HCC, especially those resistant to Sorafenib, a first generation of TKIs.^[^ ^14^, ^15^ ^]^ However, development of drug resistance to TKIs is a significant challenge limiting the clinical efficacy, leading to disease progression and treatment failure.^[^ ^16^ ^]^ The mechanisms underlying resistance to TKIs are highly complex, involving multiple cancer‐intrinsic cell signaling pathways^[^ ^17^ ^]^ and microenvironmental cues, which may converge to dysregulate the TGF‐β signaling pathway.^[^ ^18^ ^]^ Indeed, hyperactivation of TGF‐β signaling plays a significant role in mediating resistance of HCC cells to TKIs including Sorafenib, Lenvatinib, and Regorafenib.^[^ ^16^, ^17^, ^18^, ^19^, ^20^, ^21^, ^22^ ^]^ However, not all cancers are equally regulated by TGF‐β signaling. A subpopulation of CD90^+^ CSCs with hyperactive TGF‐β signaling are empowered by TGF‐β released from cancer‐associated fibroblasts in the tumor microenvironment.^[^ ^23^, ^24^ ^]^ However, it remains unclear how cancer‐intrinsic feedback regulators in the TGF‐β signaling pathway may contribute to cancer stemness and TKI resistance of HCC cells.

By performing a genome‐wide CRISPR/dCas9 activation screen using drug‐resistant CSC properties as a readout, we have identified the TGF‐β receptor‐associated binding protein 1 (TGFBRAP1 as a TGF‐β‐inducible adaptor protein to promotes activation of the TGF‐β signaling pathway in a positive feedback manner. Potentiation of the TGF‐β signaling pathway by TGFBRAP1 leads to impaired sensitivity to TKIs via enhancing stemness of HCC cells. Mechanistically, TGFBRAP1 stabilizes the TGFΒR1 by competing with the E3 ubiquitin ligases Smurf1/2 for binding to TGFBR1, thus preventing its poly‐ubiquitination and proteasomal degradation. Moreover, blocking this feedback activation of TGF‐β signaling by TGFBRAP1 increased cancer cell sensitivity to TKIs via decreasing CSC stemness, highlighting a drug combination regimen that is potentially translatable to eliminate CSCs in human cancers.

## 2 Results

### 2.1 Genome‐Wide CRISPR Activation Screen Identifies TGFBRAP1 As a Cancer Cell‐Intrinsic Regulator of Drug‐Resistant Stem‐Like Properties

**Figure 1:**
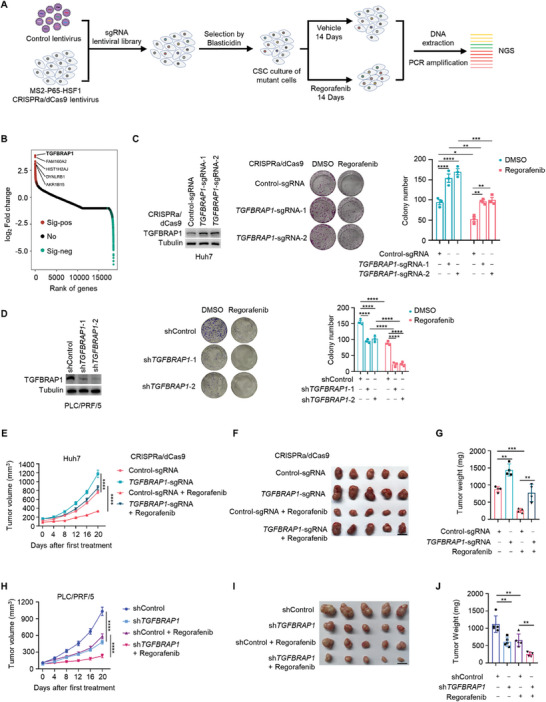
CRISPR activation screen identifies TGFBRAP1 as a candidate regulator of Regorafenib resistance. A) Schematic of the pooled CRISPR library screen. B) Sequencing results from screen of sgRNAs were sorted by the enrichment score based on Log2 (fold change). C) Western blotting analysis of lysates of Huh7 cells infected lentivirus carrying a CRISPRa‐sdCas9 activation system and control or *TGFBRAP1*‐targeting sgRNA to induce moderate overexpression of TGFBRAP1. Representative images (middle) and quantitative results (right) of colony formation assays were shown. The cells were treated with DMSO or Regorafenib (10 µM) to evaluate drug sensitivity. D) Western blotting analysis to validate the knockdown efficacy of shRNA targeting *TGFBRAP1* in PLC/PRF/5 cells (left). Representative images (middle) and quantitative results (right) of colony formation assays were shown, with cells treated with or without Regorafenib (10 µM). E) Growth curves of xenografts generated by the control or TGFBRAP1‐overexpressing Huh7 cells in nude mice that were treated with vehicle or Regorafenib (20 mg k^−1^g). F, G) Representative xenograft tumors at endpoint (F) and quantification of tumor weights in each group (G). H) Growth curves of xenografts derived from control‐ or *TGFBRAP1*‐depleted PLC/PRF/5 cells in nude mice treated with vehicle or Regorafenib (20 mg k^−1^g). I, J) Representative xenograft tumors at endpoint (I) and quantification of tumor weights in each group (J). Scale bar represents 1 cm. Statistical analyses were performed by two‐way ANOVA with Bonferroni's multiple comparisons test. *p < 0.05, **p < 0.01, ***p < 0.001 and ****p < 0.0001.

To identify potential candidates mediating drug resistance and cancer stemness, we carried out an unbiased screen by stably expressing the MS2‐P65‐HSF1 activator helper complex in the HCC cell line Huh7, followed by transduction of a pooled genome‐wide CRISPR activation sgRNA library at a low MOI (**Figure** **1**A).^[^ ^25^, ^26^ ^]^ Following selection with the TKI Regorafenib or the solvent in the CSC culture medium for two weeks, genomic DNA was extracted and subjected to next‐generation sequencing and analysis. Among the enriched genes, *TGFBRAP1* was selected for further evaluation for two reasons: first, it ranked as the top gene in which two out of three sgRNAs were significantly enriched (Figure 1B; Figure S1A, B, Supporting Information); and second, its potential role in regulating the TGF‐β signaling pathway,^[^ ^27^, ^28^ ^]^ suggesting that TGFBRAP1 might link TGF‐β signaling to drug‐resistant CSCs.

To validate these findings, HCC cells were engineered to induce moderate overexpression of TGFBRAP1 at physiological levels using a CRISPRa‐dCas9 activation system. Elevating endogenous TGFBRAP1 expression increased colony formation as well as promoted cellular growth in the presence of Regorafenib (Figure 1C; Figure S1C, Supporting Information). Conversely, depletion of *TGFBRAP1* inhibited cell growth and rendered them more sensitive to Regorafenib (Figure 1D). In vivo xenograft tumor assays demonstrated that TGFBRAP1‐overexpressing cells displayed increased tumorigenic capacity and were less sensitive to Regorafenib treatment (Figure 1E–G). Moreover, the tumor growth and Regorafenib resistance were reduced in *TGFRBAP1*‐silenced cells (Figure 1H–J). These results indicated that TGFBRAP1 promotes cell growth and confers resistance of HCC cells to Regorafenib. Additionally, *TGFBRAP1*‐depleted cells were also more sensitive to other TKIs, including Sorafenib and Lenvatinib (Figure S1D, Supporting Information), suggesting that TGFBRAP1 may be a key regulator of resistance to TKIs in liver cancer.

**Figure 2:**
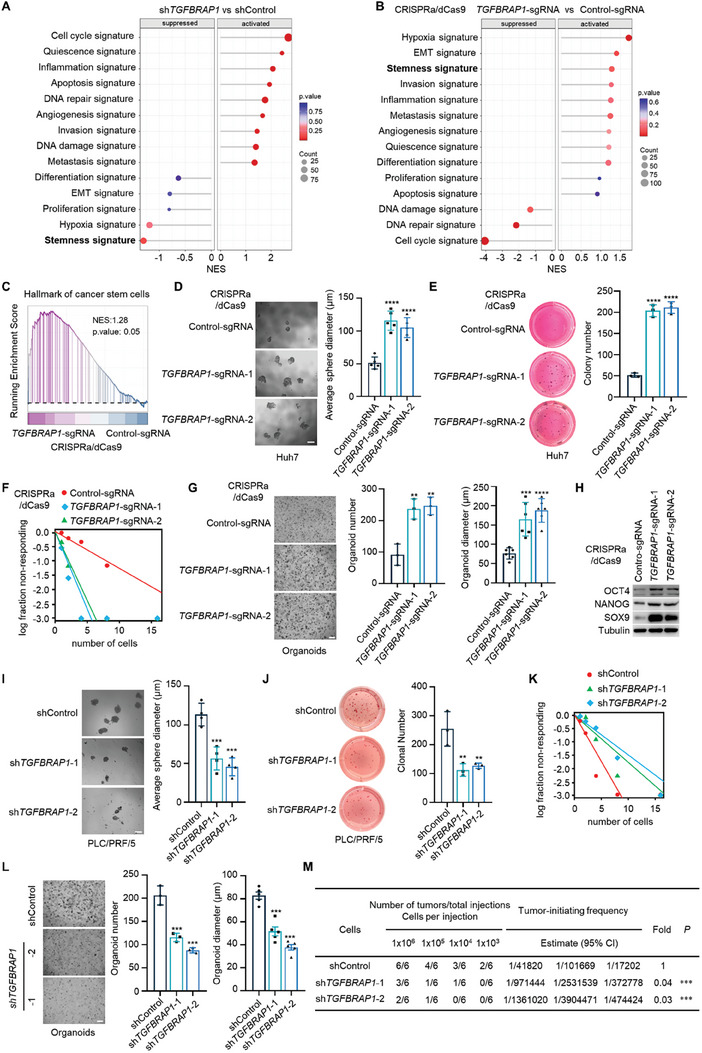
TGFBRAP1 maintains stemness of HCC cells. A, B) Gene set enrichment analysis (GSEA) of *TGFBRAP1*‐depleted cells (A) and *TGFBRAP1*‐overexpressing cells (B). C) The hallmark of cancer stem cells was enriched in TGFBRAP1‐overexpressing cells, as compared to control cells, calculated by gene signatures from MSigDb database and GSEA analysis. D) Representative images of spheres formed by control or *TGFBRAP1*‐overexpressing Huh7 cells and quantification of sphere diameter. Scale bar represents 100 µm. E) Representative images of soft agar colony formation assays of control or *TGFBRAP1*‐overexpressing Huh7 cells and quantification of colony number. F) In vitro limiting dilution assay of control or *TGFBRAP1*‐overexpressing Huh7 cells. G) Representative images of control or *TGFBRAP1*‐overexpressing HCC organoids and quantification of organoid number and diameter. Scale bar represents 500 µm. H) Western blotting analyses of OCT4, NANOG, and SOX9 in control and *TGFBRAP1*‐overexpressing Huh7 cells. I) Representative images of spheres formed by control or *TGFBRAP1*‐depleted PLC/PRF/5 cells and quantification of sphere diameter. Scale bar represents 100 µm. J) Representative images of soft agar colony formation assays of control and *TGFBRAP1*‐depleted PLC/PRF/5 cells and quantification of colony number. K) In vitro limiting dilution assay of control and *TGFBRAP1*‐depleted PLC/PRF/5 cells. L) Representative images of control and *TGFBRAP1*‐depleted HCC organoids and quantification of organoid number and diameter. Scale bar represents 500 µm. M) Frequencies of cancer‐initiating cells (CICs) of indicated PLC/PRF/5 cells were analyzed by extreme limiting dilution assays in NOD/SCID mice. Statistical analyses were performed by one‐way ANOVA comparisons test. *p < 0.05, **p < 0.01, ***p < 0.001 and ****p < 0.0001.

Transcriptome analysis of TGFBRAP1‐manipulated cells revealed that cancer stemness was among the enriched cancer hallmark signatures (**Figure** **2**A,B). CSC signatures were downregulated in *TGFBRAP1*‐depleted cells and upregulated in TGFBRAP1‐overexpressing cells, as compared to their respective control cells (Figure 2A–C). Moreover, both transcript and protein levels of TGFBRAP1 were elevated in CSC spheres derived from Huh7 and PLC/PRF/5 cells (Figure S2A, B, Supporting Information). These results suggest that TGFBRAP1 may regulate CSC stemness properties to promote drug resistance. Consistently, tumor sphere formation assays, soft agar colony formation assays, and in vitro limiting dilution assays demonstrated that TGFBRAP1‐overexpressing cells exhibited increased self‐renewal potential (Figure 2D–F; Figure S2C, Supporting Information). Moreover, ectopic expression of TGFBRAP1 in patient‐derived organoids led to a considerable increase in their number and size (Figure 2G). Additionally, the CSC markers OCT4, NANOG and SOX9, were upregulated in TGFBRAP1‐overexpressing cells (Figure 2H). On the other hand, sphere formation, soft agar colony formation, and limiting dilution sphere formation potential were inhibited in *TGFBRAP1*‐depleted cells (Figure 2I–K). The number and size of *TGFBRAP1*‐depleted organoids were also markedly reduced (Figure 2L). CSCs were endowed with enhanced invasiveness, and depletion of *TGFBRAP1* inhibited their migration and invasion abilities (Figure S2D, Supporting Information). Importantly, depleting *TGFBRAP1* led to a decrease in the frequency of cancer‐initiating cells in vivo (Figure 2M). Conversely, ectopic expression of TGFBRAP1 resulted in an expansion of cancer‐initiating cell pool in vivo (Figure S2E, Supporting Information). Thus, TGFBRAP1, a regulator of the TGF‐β signaling pathway, acts as a key driver of stem cell‐like properties and TKI resistance in HCC cells.

### 2.2 TGFBRAP1 Promotes Activation of the TGF‐β Signaling Pathway through Stabilizing TGFBR1

**Figure 3:**
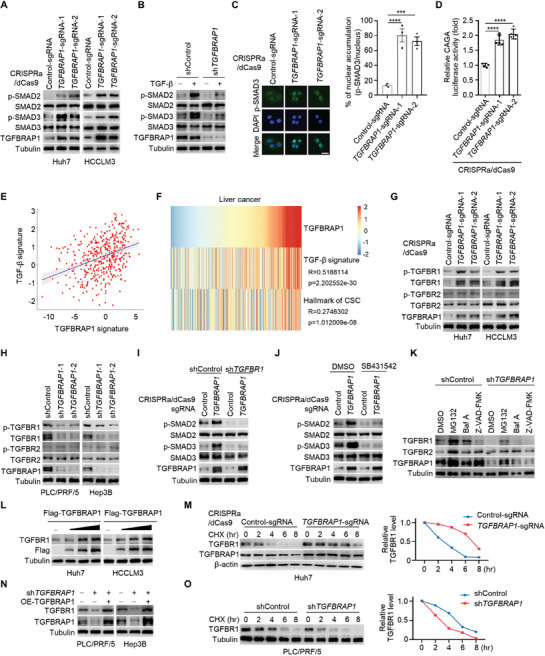
TGFBRAP1 promotes activation of the TGF‐β signaling pathway through stabilizing TGFBR1. A) Western blotting analysis of p‐SMAD2 and p‐SMAD3 levels in control and *TGFBRAP1*‐overexpressing Huh7 and HCCLM3 cells. B) Control and *TGFBRAP1*‐depleted PLC/PRF/5 cells were stimulated with TGF‐β (20 ng ml^−1^) for 4 hours. Western blotting analysis was performed as in (A) to detect TGF‐β signaling activity. C) Immunofluorescence to detect p‐SMAD3 in control and *TGFBRAP1*‐overexpressing Huh7 cells. (blue, nuclei), (green, p‐SMAD3). Scale bar represents 50 µm. D) Luciferase assay of TGF‐β‐responsive CAGA‐directed reporter in control and *TGFBRAP1*‐overexpressing Huh7 cells. E) Correlation analysis between TGFBRAP1 and TGF‐β activation signatures in TCGA HCC dataset. F) Correlation analysis of TGFBRAP1 expression levels and the TGF‐β activation signatures, as well as cancer stemness hallmark signatures, in TCGA HCC dataset. G, H) Western blotting analysis of indicated proteins in control and TGFBRAP1‐overexpressing cells (G) and in control and *TGFBRAP1* depleted cells (H). I) Western blotting analysis of p‐SMAD2 and p‐SMAD3 in indicated cells. J) Western blotting analysis of p‐SMAD2 and p‐SMAD3 in control and TGFBRAP1‐overexpressing Huh7 cells treated with DMSO or SB431542 (10 µM). K) Western blotting analysis of TGFBR1, TGFBR2, and TGFBRAP1 in control and *TGFBRAP1* depleted PLC/PRF/5 cells treated with MG132 (20 µM), Baf A (20 µM), or Z‐VAD‐FMK (20 µM) for 24 hours. L) HCC cells were transfected with increasing amounts of Flag‐TGFBRAP1 plasmid (0.5 µg, 1 µg, and 2 µg) for 48 hours and western blotting analysis of TGFΒR1 and Flag. M) Control and TGFBRAP1‐overexpressing Huh7 cells were treated with cycloheximide (CHX) for indicated time points. Western blotting analysis to detect the half‐life of TGFΒR1 (left) and quantification of staining intensity (right). N) Western blotting analysis of TGFΒR1 and TGFBRAP1 in control and *TGFBRAP1* depleted, or in *TGFBRAP1* depleted cells reconstituted with TGFBRAP1 HCC cells. O) Control and *TGFBRAP1*‐depleted PLC/PRF/5 cells treated with CHX for indicated time points. Western blotting analysis to detect the half‐life of TGFΒR1 (left) and quantification of staining intensity (right). (H). Statistical analyses were performed by one‐way ANOVA comparisons test. *p < 0.05, **p < 0.01, ***p < 0.001 and ****p < 0.0001.

Although TGFBRAP1 was reported to promote the TGF‐β signaling activity,^[^ ^27^, ^28^ ^]^ the underlying molecular basis and pathological relevance has remained largely elusive. To verify a regulatory role of TGFBRAP1 on TGF‐β signaling, the activation of TGF‐β signaling was detected by immunoblotting. Enhanced expression of endogenous TGFBRAP1 promoted phosphorylation of SMAD2 and SMAD3 at basal levels (**Figure** **3**A). Meanwhile, p‐SMAD2/3 levels were decreased in *TGFBRAP1*‐depleted cells following TGF‐β stimulation (Figure 3B). Activation of TGF‐β signaling is associated with nuclear entry of p‐SMAD3 to promote transcription of target genes. Consistently, elevating endogenous TGFBRAP1 expression enhanced nuclear localization of p‐SMAD3, leading to upregulation of CAGA‐luciferase reporter activity (Figure 3C,D), a robust indicator of transcriptional activity mediated by the TGF‐β signaling pathway.^[^ ^29^ ^]^ To gain pathological insights, a TGFBRAP1‐regulated gene signature was drawn by analyzing the transcriptomic profile of TGFBRAP1‐overexpressing cells. We observed that the TGFBRAP1 signature, as well as the TGFBRAP1 expression status, correlated with the TGF‐β signaling activity in human HCC tissues (Figure 3E,F). Moreover, high levels of TGFBRAP1 expression are associated with the CSC stemness signature in HCC (Figure 3F). Thus, TGFBRAP1‐mediated activation of TGF‐β signaling is pathologically relevant to human HCC.

We sought to dissect how TGFBRAP1 promotes the activation of the TGF‐β signaling pathway. Interestingly, p‐SMAD2/3 levels were regulated in TGFBRAP1‐manipulated cells (Figure 3A,B), suggesting a unique function of TGFBRAP1 that may be distinct from its previously reported role as a SMAD4 chaperone.^[^ ^24^ ^]^ These results also suggest that TGFBRAP1 may act upstream of SMAD2/3 phosphorylation, which are directly phosphorylated by TGFBR1.^[^ ^3^ ^]^ We thus examined whether TGFBRAP1 might regulate either TGFΒR1 or TGFBR2 receptors, and observed that upregulation of endogenous TGFBRAP1 led to an increased abundance of total and phosphorylated TGFΒR1, with minimal effects on TGFBR2 (Figure 3G). Conversely, depletion of *TGFBRAP1* decreased total and phosphorylated TGFBR1 levels (Figure 3H). These results indicate that TGFBRAP1 may upregulate TGFBR1 to promote downstream SMAD2/3 signaling. Consistent with this notion, TGFBRAP1‐mediated activation of SMAD2/3 signaling was attenuated in *TGFΒR1*‐depleted cells (Figure 3I). This phenotype was recapitulated by a TGFΒR1 selective inhibitor SB431542 (Figure 3J). Conversely, ectopic expression of TGFBR1 rescued TGF‐β signaling activity in *TGFBRAP1*‐depleted cells (Figure S3A, Supporting Information). Reminiscent of the role of TGFBRAP1 in maintaining drug‐resistant stem‐like properties, depletion of *TGFBR1*, the TGFBRAP1‐interactor, also decreased stemness properties, leading to increased sensitivity to TKIs (Figure S3B–D, Supporting Information). These results suggest that TGFBRAP1 promotes SMAD2/3 signaling activity via upregulating TGFBR1.

It is noteworthy that TGFΒR1 mRNA remained unchanged upon depletion or overexpression of TGFBRAP1 (Figure S3E, F, Supporting Information), indicating that TGFBRAP1 might regulate TGFΒR1 at the post‐transcriptional levels. Moreover, TGFΒR1 expression was rescued by MG132, an inhibitor of the 26S proteasome, suggesting that TGFΒR1 was primarily degraded in a proteasomal‐dependent manner (Figure 3K). Consistently, ectopic expression of TGFBRAP1 upregulated TGFΒR1 expression by prolonging its protein half‐life (Figure 3L,M). On the other hand, depleting *TGFBRAP1* shortened the half‐life of TGFΒR1 in cells (Figure 3N,O). These findings suggest that TGFBRAP1 promotes the TGF‐β signaling pathway activity largely by stabilizing TGFBR1 protein.

### 2.3 TGFBRAP1 Protects TGFBR1 from Undergoing Poly‐Ubiquitination and Proteasomal Degradation by Competition with the E3 Ubiquitin Ligases Smurf1/2

**Figure 4:**
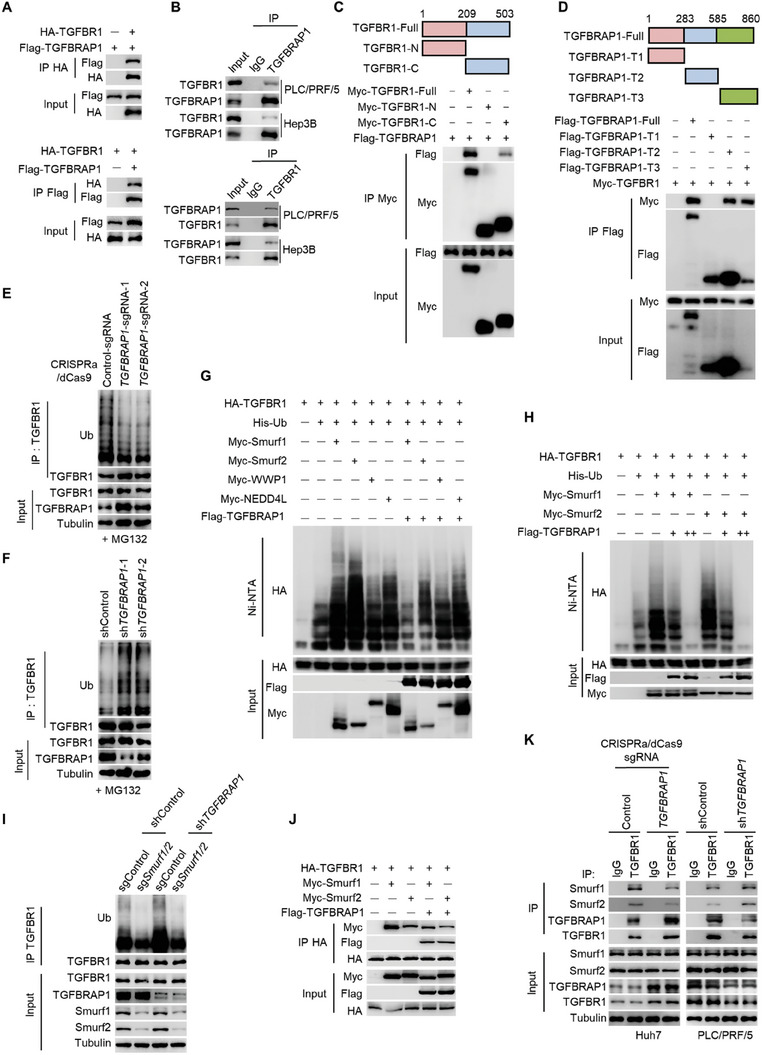
TGFBRAP1 protects TGFBR1 from poly‐ubiquitination and proteasomal degradation by competing with the E3 ubiquitin ligases Smurf1/2. A) Co‐immunoprecipitation (Co‐IP) analysis of association between exogenous HA‐TGFΒR1 and Flag‐TGFBRAP1 in HEK293T cells. B) Co‐IP analysis of association between endogenous TGFBRAP1 and TGFΒR1 in HCC cells. C) HEK293T cells were transfected with indicated Myc‐tagged TGFΒR1 truncations together with Flag‐tagged TGFBRAP1 for 48 hours. Input and co‐IP were detected by western blotting analysis. D) HEK293T cells were transfected with indicated Flag‐tagged TGFBRAP1 truncations together with Myc‐tagged TGFΒR1 for 48 hours. Input and co‐IP were detected by western blotting analysis. E, F) Immunoprecipitation and western blotting analysis to detect ubiquitination of endogenous TGFΒR1 in TGFBRAP1‐overexpressing Huh7 cells (E) and *TGFBRAP1* depleted PLC/PRF/5 cells (F). G) HEK293T cells were transfected with indicated plasmids for 48 hours. Immunoprecipitation and western blotting analysis to detect ubiquitination of exogenous TGFΒR1. H) HEK293T cells were transfected with indicated plasmids or increasing amounts of Flag‐tagged TGFBRAP1 plasmid for 48 hours. Immunoprecipitation and western blotting analysis to detect the ubiquitination of exogenous TGFΒR1. I) Smurf1 and Smurf2 were depleted in control and TGFBRAP1 depleted PLC/PRF/5 cells. Immunoprecipitation and western blotting analysis to detect the ubiquitination of endogenous TGFΒR1. J) HEK293T cells were transfected with indicated plasmids for 48 hours. Co‐IP and western blotting analysis to detect the interaction between TGFΒR1 and Smurf1/2. K) The association of TGFΒR1 with Smurf1 and Smurf2 was detected by Co‐IP and western blotting analysis in control and TGFBRAP1‐overexpressing Huh7 cells (left) and in control and *TGFBRAP1* depleted PLC/PRF/5 cells (right).

To identify how TGFBRAP1 stabilizes the TGFΒR1 protein, we found that ectopically expressed TGFBRAP1 and TGFΒR1 interacted in HEK293T cells (**Figure** **4**A). The mutual interactions of TGFBRAP1 and TGFΒR1 were also confirmed in HCC cell lines at endogenous levels (Figure 4B). Domain mapping by co‐immunoprecipitation assays identified that the C‐terminal region of TGFΒR1 and the T2 and T3 domains of TGFBRAP1 were mediating the interaction between these two proteins (Figure 4C,D). As such, a TGFBRAP1 mutant lacking the T2 and T3 domains, and therefore unable to interact with TGFBR1, was unable to promote SMAD2/3 signaling, leading to impaired stemness properties of HCC cells (Figure S4A–C, Supporting Information).

Since TGFΒR1 was primarily degraded via the 26S proteasome, we examined whether TGFBRAP1 could affect the ubiquitin levels of TGFBR1. We observed that upregulating TGFBRAP1 expression reduced poly‐ubiquitination levels of TGFBR1, while depleting *TGFBRAP1* led to an increase in the poly‐ubiquitination status of TGFΒR1 (Figure 4E,F). These findings suggest that TGFBRAP1 interacts with TGFΒR1 to inhibit its poly‐ubiquitination, thereby stabilizing TGFΒR1 to promote TGF‐β signaling activity.

TGFBRAP1 is neither an E3 ubiquitin ligase nor a deubiquitinase, thus its role in regulating protein stability of TGFBR1 may be indirect. Smurf1, Smurf2, WWP1, and NEDD4L are putative E3 ubiquitin ligases of TGFΒR1.^[^ ^30^, ^31^, ^32^, ^33^ ^]^ To define how TGFBRAP1 suppresses the ubiquitination of TGFΒR1, we examined whether TGFBRAP1 could regulate the ubiquitination of TGFΒR1 mediated by any of these E3 ubiquitin ligases. The ubiquitination of TGFΒR1 mediated by Smurf1 and Smurf2 was decreased in the presence of TGFBRAP1, whereas the ubiquitination level of TGFΒR1 mediated by WWP1 and NEDD4L was unchanged (Figure 4G). Furthermore, TGFBRAP1 could inhibit the ubiquitination of TGFΒR1 mediated by Smurf1/2 in a dose‐dependent manner (Figure 4H). Meanwhile, following depletion of *TGFBRAP1*, we observed an elevation of TGFΒR1 ubiquitination which was reversed when *Smurf1/2* were co‐depleted (Figure 4I). Conversely, overexpression of TGFBRAP1 reduced ubiquitination levels of TGFΒR1 induced by Smurf1/2 co‐expression (Figure S4D, Supporting Information).

We next investigated whether TGFBRAP1 could affect the binding of Smurf1/2 to TGFΒR1. Ectopic expression of TGFBRAP1 reduced the interaction between TGFΒR1 and Smurf1/2 in HEK293T cells (Figure 4J). Likewise, overexpression of TGFBRAP1 in HCC cells reduced the interaction between Smurf1/2 and TGFBR1 (Figure 4K). Meanwhile, the binding of Smurf1/2 to TGFΒR1 was increased in *TGFBRAP1*‐depleted cells (Figure 4K). Consequently, ectopic expression of TGFBRAP1 partially attenuated down‐regulation of TGFΒR1 induced by Smurf1/2 (Figure S4E, Supporting Information). These results demonstrate that TGFBRAP1 competes with Smurf1/2 for binding to TGFΒR1, thereby inhibiting Smurf1/2‐mediated TGFΒR1 poly‐ubiquitination and proteasomal degradation.

### 2.4 *TGFBRAP1* is Transcriptionally Up‐Regulated by the TGF‐β/SMAD Signaling Pathway in a Positive Feedback Manner

**Figure 5:**
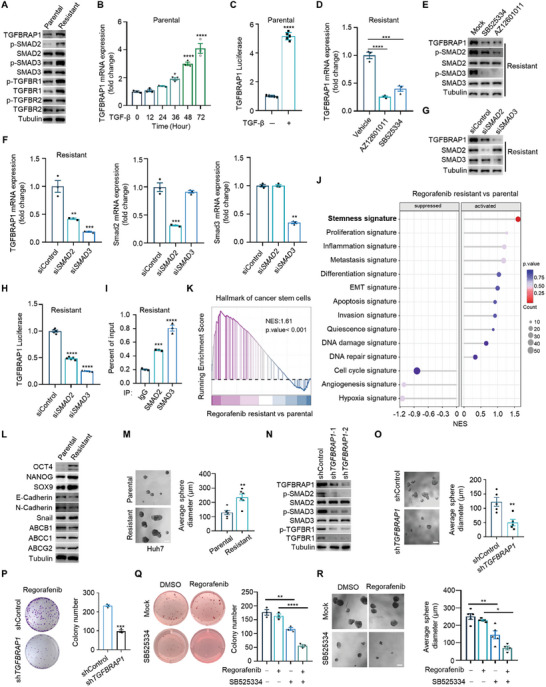
TGFBRAP1 is transcriptionally up‐regulated by the TGF‐β/SMAD signaling pathway in a positive feedback manner. A) Western blotting analysis of TGFBRAP1 and components of the TGF‐β signaling pathway in parental cells and Regorafenib‐resistant counterparts which was established via prolonged culture of Huh7 cells with Regorafenib for more than six months. B) Parental Huh7 cells were treated with TGF‐β for indicated time points. TGFBRAP1 mRNA was detected by quantitative PCR. C) Parental Huh7 cells were transfected with TGFBRAP1 promoter luciferase reporter plasmid and treated with TGF‐β for 24 hours. D) Quantitative PCR analysis of TGFBRAP1 mRNA expression in Regorafenib‐resistant Huh7 cells treated with AZ12601011 or SB525334. E) Western blotting analysis to detect TGFBRAP1 expression and TGF‐β signaling activity in Regorafenib‐resistant Huh7 cells treated with SB525334 or AZ12601011. F,G) Expression of TGFBRAP1, SMAD2, and SMAD3 were determined in Regorafenib‐resistant Huh7 cells transfected with *SMAD2* or *SMAD3* siRNA. Quantitative PCR assay of mRNA (F) and western blotting analysis of protein lysates (G). H) TGFBRAP1 promoter luciferase in Regorafenib‐resistant Huh7 cells following depletion of *SMAD2* or *SMAD3* using siRNA. I) ChIP analysis of SMAD2/3 occupancy in the promoter of *TGFBRAP1* in Regorafenib‐resistant Huh7 cells. J) Analysis of cancer hallmark signatures in the transcriptome of Regorafenib‐resistant and parental Huh7 cells. K) GSEA analysis of the cancer stem cell signature in Regorafenib‐resistant and parental Huh7 cells. L) Western blotting analysis of indicated proteins in the Regorafenib‐resistant and parental Huh7 cells. M) Sphere formation assays of Regorafenib‐resistant and parental Huh7 cells. Scale bar represents 100 µm. N) Western blotting analysis of TGFBRAP1 and TGF‐β signaling in the Regorafenib‐resistant Huh7 cells transfected with control or *TGFBRAP1* shRNA. O) Sphere formation assays of Regorafenib‐resistant Huh7 cells transfected with control or *TGFBRAP1* shRNA. Scale bar represents 100 µm. P) Colony formation assays of Regorafenib‐resistant Huh7 cells transfected with control or *TGFBRAP1* shRNA. Representative images of colony formation assays (left) and quantification of colony number (right). Q) Soft agar colony formation assays of Regorafenib‐resistant Huh7 cells treated with indicated drugs. R) Sphere formation assay of Regorafenib‐resistant Huh7 cells treated by indicated drugs. Scale bar represents 100 µm. Statistical analyses were performed by Student's t‐test or one‐way ANOVA with Bonferroni's multiple comparisons test. *p < 0.05, **p < 0.01, ***p < 0.001 and ****p < 0.0001.

Given a previously unrecognized role of TGFBRAP1 in stabilizing TGFΒR1, we next assessed how the expression of TGFBRAP1 is regulated. Unexpectedly, in analyzing a drug‐resistant cell line that underwent prolonged selection with Regorafenib for over six months, TGFBRAP1 expression was found to be markedly up‐regulated in the resistant cells as compared to the parental counterparts (**Figure** **5**A). Similarly, TGFBRAP1 expression was upregulated in Sorafenib‐resistant HCC cells (Figure S5A, B, Supporting Information). Furthermore, the drug‐resistant cells displayed hyperactivation of the TGF‐β signaling pathway, as well as elevated levels of total and phosphorylated TGFΒR1 (Figure 5A). These findings indicate that TGFBRAP1 expression might be under the control of the TGF‐β signaling pathway in the TKI‐resistance cells.

Supporting this hypothesis, mRNA levels of TGFBRAP1 in parental cells were induced by TGF‐β stimulation in a time‐dependent manner (Figure 5B). By employing a dual luciferase reporter assay, we observed that the promoter activity of TGFBRAP1 was remarkably enhanced by TGF‐β (Figure 5C), suggesting that TGF‐β signaling transcriptionally up‐regulates *TGFBRAP1*. Consistently, inhibition of TGFBR1 by AZ12601011 and SB525334 significantly down‐regulated TGFBRAP1 mRNA and protein levels in the drug‐resistant cells (Figure 5D,E). Moreover, depletion of *SMAD2* and *SMAD3* by small interfering RNAs also reduced TGFBRAP1 expression in these cells (Figure 5F,G). A binding site for SMAD2/3 was also identified in the *TGFBRAP1* promoter sequence. By inserting this binding motif in a luciferase reporter, we observed that depletion of *SMAD2* or *SMAD3* significantly inhibited transcriptional activity of the *TGFBRAP1* promoter in the drug‐resistant cells (Figure 5H). Chromatin immunoprecipitation (ChIP) analysis further confirmed recruitment of SMAD2/3 on the promoter of *TGFBRAP1* (Figure 5I). Collectively, TGFBRAP1 undergoes positive feedback regulation by the TGF‐β/SMAD signaling pathway, leading to its up‐regulation in TKI‐resistant cells.

We next questioned whether this feedback regulation was related to cancer stemness and drug resistance. Transcriptome analysis revealed that the TKI‐resistant cells were highly enriched with cell signaling activity linked to cancer stemness, as compared to the parental cells (Figure 5J,K). Expression of CSC markers were elevated in the TKI‐resistant cells as well, while other EMT and multidrug resistance markers were partially altered (Figure 5L; Figure S5C–E, Supporting Information). Functionally, drug‐resistant cells exhibited a higher self‐renewing capacity, as indicated by tumor sphere formation assays, which is consistent with their enhanced TGF‐β signaling (Figure 5M). Whereas depletion of *TGFBRAP1* in these TKI‐resistant cells down‐regulated the expression level of TGFΒR1, leading to suppression of downstream TGF‐β/SMAD signaling pathway and attenuated stem‐like growth capacity (Figure 5N,O). These results highlight an essential role for this TGFBRAP1 positive feedback loop in further promoting TGF‐β signaling activity to sustain stemness of TKI‐resistant cells.

Consistent with impaired TGF‐β signaling activity and cancer stemness, *TGFBRAP1*‐depleted resistant cells exhibited increased sensitivity to Regorafenib (Figure 5P). Pharmacological inhibition of TGFΒR1 also attenuated their resistance capacity, leading to reduced colony formation in soft agar and sphere formation in the presence of Regorafenib (Figure 5Q,R). Moreover, we observed that depletion of *TGFBRAP1* in the resistant cells sensitized the xenograft tumors to Regorafenib in nude mice (Figure S5F–H, Supporting Information). Collectively, transcriptional upregulation of *TGFBRAP1* by TGF‐β signaling hyperactivates the signaling pathway in a positive feedback loop and maintains stem‐like drug‐resistant properties of HCC cells.

### 2.5 High Expression Levels of the TGFBRAP1‐TGFBR1 Reciprocal Loop are Associated with Malignant Progression of HCC

**Figure 6:**
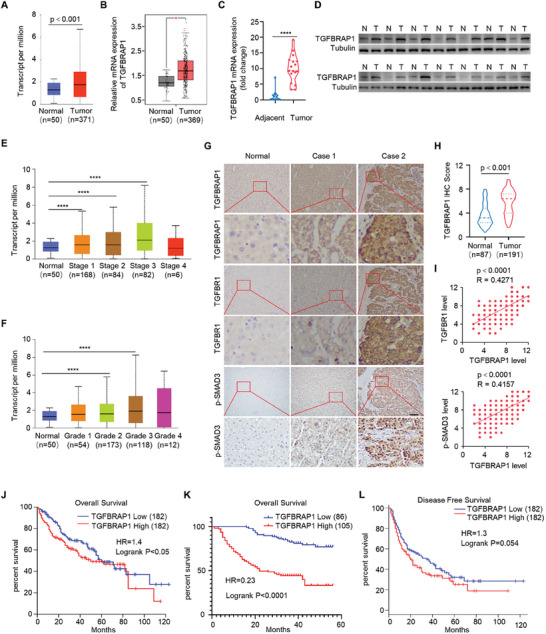
High expression of TGFBRAP1 is associated with HCC malignancy and clinical prognosis. A) TGFBRAP1 expression from UALCAN TCGA database. B) TGFBRAP1 expression from GEPIA TCGA database. C) mRNA expression of TGFBRAP1 in HCC patients, n = 17. D) Western blotting analysis of TGFBRAP1 protein in HCC patients. N, non‐tumor tissue. T, tumor tissue. E, F) Expression of TGFBRAP1 from UALCAN TCGA database in indicated advanced clinical stage (E) and tumor grade (F). G) Representative immunohistochemical (IHC) images of HCC and normal tissues. Scale bar represents 200 µm. H) The IHC score of TGFBRAP1 in normal and tumor tissues. I) Protein level correlation of TGFBRAP1, TGFBR1 and p‐SMAD3. J–L) Kaplan‐Meier survival analysis shows that high expression of TGFBRAP1 in HCC tissues correlates with shortened overall survival (J) and disease‐free survival (L) by analyzing dataset from GEPIA database, and reduced overall survival (K) by analyzing an in‐house dataset from Daping Hospital. *p < 0.05, **p < 0.01, ***p < 0.001 and ****p < 0.0001.

We next assessed whether the reciprocal regulation of TGFBRAP1 and TGF‐β signaling is relevant to human HCC. In support of this notion, analysis of datasets from TCGA from UALCAN (ualcan.path.uab.edu/) (**Figure** **6**A), and GEPIA (http://gepia.cancer‐pku.cn/) (Figure 6B), revealed that mRNA expression levels of *TGFBRAP1* were elevated in HCC tissues. These results were validated by qRT‐PCR analysis of an in‐house HCC tissue cohort (Figure 6C). The protein levels of TGFBRAP1 were also increased by immunoblotting analysis of freshly collected HCC specimens relative to adjacent non‐tumor tissues (Figure 6D). By examining a larger cohort of more than 300 HCC tissues, *TGFBRAP1* expression levels were positively correlated with clinical parameters such as advanced clinical stage and tumor grade (UALCAN, ualcan.path.uab.edu/) (Figure 6E,F). Thus, we conclude that TGFBRAP1 expression is increased in HCC tissues and tightly associated with disease progression.

Moreover, TGFBRAP1 expression status was tightly associated with expression levels of TGFΒR1 and the activity of TGF‐β signaling in HCC tissues. Analysis of immunohistochemical staining scores revealed that the expression level of TGFBRAP1 was higher in HCC tissues than in matched normal liver tissues (Figure 6G,H). HCC tissues with high levels of TGFBRAP1 expression were associated with larger tumor size and higher histological grades (Table S1, Supporting Information). Importantly, the staining scores of TGFΒR1 and p‐SMAD3 were both higher in samples with increased levels of TGFBRAP1 expression (Figure 6G,I), further indicating that TGFBRAP1 may upregulate expression of TGFΒR1 to promote the TGF‐β signaling pathway during human liver tumorigenesis. As such, compared to patients with TGFBRAP1^low^ tumors, those with TGFBRAP1^high^ tumors showed poorer overall survival (Figure 6J–L). Together, these data indicated that the TGFBRAP1‐TGFBR1 regulatory loop is activated in human HCC and correlates with disease progression.

### 2.6 Targeting TGFΒR1 Enhances the Efficacy of Regorafenib via Decreasing CSCs

**Figure 7:**
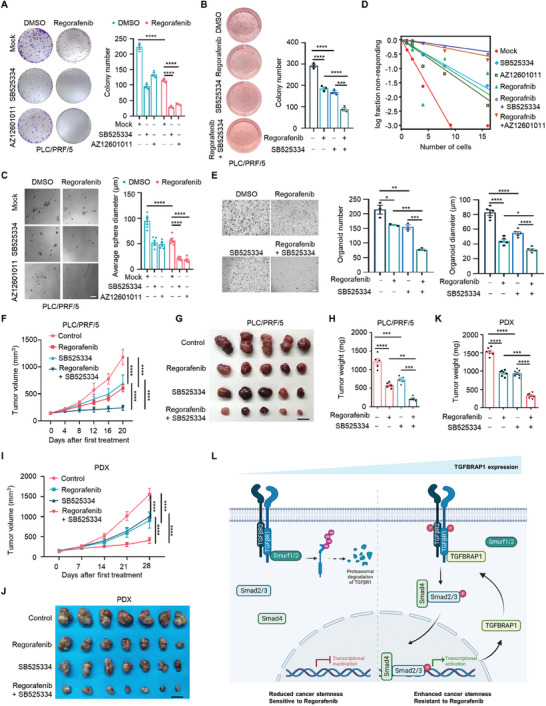
Targeting TGFΒR1 enhances the efficacy of Regorafenib via deceasing CSCs. A) Colony formation assays of PLC/PRF/5 cells treated with Regorafenib (10 µM), SB525334 (10 µM) or AZ12601011 (10 µM) alone or in combination. Representative images of colony formation assays (left) and quantification of colony number (right). B) Soft agar colony formation assays of PLC/PRF/5 cells treated with Regorafenib or SB525334 alone or in combination. Reprssesentative images (left) and quantification of colony number (right). C) Representative images of spheres formed by PLC/PRF/5 cells treated by Regorafenib, SB525334 or AZ12601011 alone or in combination (left) and quantification of sphere diameter (right). Scale bar represents 100 µm. D) In vitro limiting dilution assay of PLC/PRF/5 cells treated as in (C). E) Representative images of HCC organoids treated with indicated drugs and quantification of organoid number and diameter. Scale bar represents 500 µm. F). Growth curves of xenografts derived from PLC/PRF/5 cells in nude mice treated with either Regorafenib (20 mg k^−1^g) or SB525334 (20 mg k^−1^g) alone or in combination. G,H) Representative xenograft tumors at endpoint (G) and quantification of tumor weights in each group (H). I) Growth curves of HCC PDX grown in NOD/SCID mice treated with either Regorafenib (20 mg k^−1^g) or SB525334 (20 mg k^−1^g) alone or in combination. J,K) Representative PDX tumors at endpoint (J) and quantification of tumor weights in each group (K). Scale bar represents 1 cm. L) Schematic model illustrating that high expression of TGFBRAP1, a feedback activator of the TGF‐β signaling pathway, promotes cancer stemness and resistance to TKIs in HCC. Statistical analyses were performed by two‐way ANOVA with Bonferroni's multiple comparisons test. *p < 0.05, **p < 0.01, ***p < 0.001 and ****p < 0.0001.

The above findings suggest that the TGFBRAP1‐TGFΒR1 feedback loop could be exploited for therapeutic purposes. Although growth of HCC cells was inhibited by Regorafenib treatment, application of TGFΒR1 inhibitors SB525334 or AZ12601011 elicited a powerful synergistic effect to retard the colony formation capacities of these cells (**Figure** **7**A,B). Moreover, combination treatment of TGFΒR1 inhibitors and Regorafenib significantly attenuated stemness of HCC cells, as revealed by tumor sphere formation and in vitro limiting dilution assays (Figure 7C,D). Similarly, TGFBR1 inhibition also sensitized the TGFBRAP1‐overexpressing cells to Regorafenib, as revealed by a strong synergistic therapeutic effect in impairing both colony and tumor sphere formation capacities (Figure S6A, B, Supporting Information). Consequently, the combination treatment demonstrated a remarkable effect in inhibiting the number and size of PDX‐derived cancer organoids, as compared to treatment with Regorafenib alone (Figure 7E).

The therapeutic activity of TGFΒR1 inhibition was further evaluated using an in vivo xenograft model. Reminiscent of our cell culture data, Regorafenib indeed inhibited growth of subcutaneous tumors in nude mice. However, inhibition of TGFΒR1 by SB525334 showed a profound combination effect with Regorafenib, leading to smaller tumor size and weight (Figure 7F–H). Consistently, combinatory administration of another TGFΒR1 inhibitor, AZ12601011, also substantially enhanced Regorafenib‐mediated inhibition on tumor growth (Figure S6C–E, Supporting Information). More importantly, TGFΒR1 inhibition also sensitized PDX‐derived xenograft tumors to Regorafenib treatment in vivo (Figure 7I–K; Figure S6F–H, Supporting Information), suggesting that this combination drug strategy may be potentially applicable to attenuate therapeutic resistance of HCC in clinical settings.

## 3 Discussion

The present study pinpoints TGFBRAP1 in promoting TGF‐β signaling at the cell surface receptor level. Using the drug‐resistant stemness phenotype as a readout for a CRISPR activation screen, we identified TGFBRAP1 as a previously under‐appreciated feedback amplifier of the TGF‐β signaling pathway. TGFBRAP1 has previously been characterized as a SMAD4 chaperone that transduces signal from the TGF‐β receptor to transcription factors.^[^ ^28^ ^]^ Deletion of *TGFBRAP1* inhibits the activity of the 3TP‐Luc reporter induced by TGF‐β.^[^ ^23^ ^]^ As a result, the phosphorylation level of SMAD2 is reduced following depletion of *TGFBRAP1*.^[^ ^34^ ^]^ These results suggest that TGFBRAP1 acts on SMADs to activate SMAD2/3 transcriptional activity. However, our data further demonstrate that TGFBRAP1 functions by interacting with TGFBR1, indicating that TGFBRAP1 associates with multiple components of the TGF‐β signaling pathway to execute its regulatory functions. Moreover, *TGFBRAP1* is transcriptionally up‐regulated by the SMAD2/3 complex, thus representing a new mode of positive feedback regulation of the TGF‐β signaling pathway. These data support the notion that TGFBRAP1, a feedback activator of the TGF‐β signaling pathway, serves as a key node at the receptor level to enhance signaling activity (Figure 7L).

These findings also elucidate a new layer of post‐transcriptional modification (PTM) controlling the abundance of the TGF‐β receptor, the first step in dictating the intracellular SMAD signaling cascade. Upon binding of TGF‐β ligands to TGFBR2 that functions as an upstream kinase of TGFBR1 at the cell membrane, TGFBR1 is activated to promote phosphorylation and activation of receptor‐associated SMAD2/3. As an important PTM, ubiquitination plays a key role in regulating stability/activity of TGFBR1, thus controlling the downstream signaling output.^[^ ^35^ ^]^ Several E3 ubiquitin ligases including Smurf1, Smurf2, WWP1, and NEDD4L, have been previously studied to target TGFBR1 for ubiquitination and degradation.^[^ ^30^, ^31^, ^32^, ^33^ ^]^ Interestingly, the inhibitory *SMAD7* is transcriptionally induced by TGF‐β ligands to recruit these E3 ligases to degrade TGFBR1.^[^ ^33^, ^36^ ^]^ Besides, a TGF‐β target LncRNA, LITATS1, could also enhance poly‐ubiquitination levels of TGFBR1 by sequestering Smurf2 in the cytoplasm.^[^ ^37^ ^]^ In contrast, here TGFBRAP1 is a positive feedback product of TGF‐β to maintain the stability of TGFBR1. Mechanistically, TGFBRAP1 competes with Smurf1 and Smurf2 to prevent their access to TGFBR1, thereby inhibiting its ubiquitination and proteasomal degradation. Collectively, multiple feedback regulators, either negative (the Smad7‐Smurf2 complex and LITATS1) or positive (TGFBRAP1), dictate homeostatic expression levels of TGF‐β receptor to modulate the strength and duration of downstream signaling. However, TGF‐β signaling has been known for its context‐dependent roles in cancers which are influenced by various factors, including the stage of cancer development, the cellular microenvironment, and the specific genetic alterations present within the cancer cells. Thus, further studies are necessary to define under which contexts each of these regulatory mechanisms are utilized to regulate TGF‐β signaling in normal and pathological settings.

Consistent with an essential role in sustaining the activation of the TGF‐β signaling pathway, TGFBRAP1 demonstrates robust activity in maintaining resistance to Regorafenib and cancer stemness of HCC cells. The TGF‐β signaling pathway plays a pivotal role in regulating CSCs and drug resistance in a series of human cancers, such as HCC, squamous cell carcinoma, colorectal cancer, glioblastoma, and triple‐negative breast cancer.^[^ ^38^, ^39^, ^40^, ^41^, ^42^, ^43^, ^44^ ^]^ Consistent with this, TGFBRAP1 expression, as well as TGF‐β signaling activity, are correlated with stem‐like properties that are highly enriched in Regorafenib‐resistance cancer cells. Consequently, depletion of *TGFBRAP1* abrogates the CSCs pool and increases sensitivity to Regorafenib.

As such, TGFBRAP1‐mediated stabilization of TGFBR1 is relevant to human liver tumorigenesis and represents a potentially therapeutic target to reverse resistance to TKIs. Up to now, a role for TGFBRAP1 in human malignancies is poorly understood. Our findings indicate that TGFBRAP1 expression is elevated in HCC tissues, and correlates with disease progression and dismal outcomes of patients. Moreover, TGFBRAP1 expression levels are tightly associated with TGFBR1, as well as activity of the TGF‐β signaling pathway, which is consistent with a critical role for TGF‐β in maintaining CSC stemness and progression of HCC. Given that the TGF‐β signaling pathway is highly activated in Regorafenib‐resistant liver cancer cells,^[^ ^45^ ^]^ targeting TGFBR1 stabilized by TGFBRAP1 could provide a novel therapeutic vulnerability. Consistent with this notion, we observed that selective inhibition of TGFBR1 renders HCC cells sensitive to Regorafenib via reducing CSCs and tumorigenicity. However, in early stages of tumorigenesis, intact TGF‐β signaling acts as a tumor suppressor by inhibiting the uncontrolled growth of the pre‐cancerous cells, which should be taken into consideration in designing therapeutic agents that targets TGFBRAP1 to inactivate the TGF‐β signaling. Taken together, blocking the TGFBRAP1 positive feedback activation of the TGF‐β signaling pathway could be a translatable strategy to alleviate resistance to multiple TKIs in late‐stage liver cancers.

## 4 Experimental Section

### Plasmids, Lentiviral Vectors, and Chemicals

Expression vectors encoding HA‐, Flag‐, Myc‐, and His‐tagged proteins were cloned into the pcDNA3.1 plasmid by inserting PCR‐amplified fragments. Promoter luciferase reporter for *TGFBRAP1* was constructed by inserting the promoter fragment PCR‐amplified from genomic DNA into the pGL3 plasmid. CAGA luciferase reporter plasmid and truncations of TGFBRAP1 or TGFBR1 were purchased from Sangon Biotech. To generate CRISPRa/dCas9 activation cell line, HCC cells were initially infected with the lentiMPH‐V2 virus to express MS2‐P65‐HSF1. Subsequently, gRNA sequence was cloned into lentiSAM V2 vector. Lentiviral shRNA vectors were generated by cloning shRNA fragments into the pLKO.1 vector. siRNAs were purchased from Sangon Biotech. The sequences of shRNA, sgRNA and siRNA were listed in Table S3 (Supporting Information). Lentiviral particles were generated through the co‐transfection of lentiviral vectors with envelope (pMD2G) and packaging (psPAX2) plasmids, into HEK293T cells using PEI transfection reagent. After 48 hours, viral containing supernatants were collected and concentrated. Chemical reagents including MG132, Bafilomycin A1, and Z‐VAD‐FMK were purchased from Selleckchem. Cycloheximide, Regorafenib, Sorafenib, Lenvatinib, SB431542, SB525334, and AZ12601011 were purchased from MedChemExpress.

### Cell Culture

Human HCC cell line (Huh7, Hep3B, and PLC/PRF5) were purchased from the Cell Bank of Shanghai Institute of Cell Biology (Shanghai, China). HCCLM3 and HEK293T were purchased from Procell Life Science and Technology (Wuhan, China). The authenticity of all cell lines were validated through short tandem repeat analysis. All cell lines were maintained in DMEM (Gibco, USA) supplemented with 10% heat‐inactivated FBS (Niological Industries, Israel), 100 units ml^−1^ penicillin, and 100 µg ml^−1^ streptomycin (Hyclone), and grown in a 5% CO_2_ humidified cell culture incubator at 37 °C.

### Pooled Synthetic Lethal CRISPR Screen

The human CRISPR 2‐plasmid activation pooled library (SAM) was purchased from Addgene (Feng Zhang lab, Addgene #1000000078)^[^ ^26^ ^]^ and applied to screen candidates mediating drug resistance and cancer stemness. Briefly, the lentiMPH v2 plasmid was stably integrated into Huh7 cell line and selected with hygromycin to obtain stable clones. The MPH‐expressing Huh7 cells were then transduced with the sgRNA activation library lentiviruses with an MOI of 0.3. Subsequently, the transduced HCC pool was selected with blasticidin to obtain a pool of successfully infected mutant cells. The mutant cells were maintained in CSC medium^[^ ^46^ ^]^ and selected with either Regorafenib (7 µM) or DMSO for 14 days. Genomic DNA was isolated from the two populations of treated cells and the sgRNA sequence was determined by the Illumina MiniSeq with custom sequencing primers based on the PCR amplified sgRNA‐encoding regions. The MAGeCK Robust was then used Rank Algorithm to analyze gRNA enrichment. A permutation test was employed to calculate the *p*‐value for each gene, subsequently adjusting for false discovery rates through the application of the Benjamini‐Hochberg method.

### Patients and HCC Tissues

Written informed consent was obtained from individual patients whose tissue and clinical data were involved. The study was approved by the Institutional Review Board of Daping Hospital. HCC and adjacent non‐tumor live tissues were obtained from 100 patients diagnosed with primary HCC who underwent curative hepatectomy at the Daping Hospital between 2015 and 2018 or purchased from Shanghai Outdo Biotech Company (HLivH180Su17). Paraffin‐embedded HCC tissues were examined by IHC and fresh specimens were collected for detection by qRT‐PCR and western blotting.

### Mouse Xenograft Tumor Model and PDXs

BALB/c nude mice (female, 6 weeks old) and NOD/SCID mice (female, 6 weeks old) were purchased from Gempharmatech China. All animal experimental procedures were approved by the Animal Ethics Committee of Army Medical University and conformed to the Guidelines for Animal Experiments of Laboratory Animals. HCC cells (1 × 10^6^) were injected subcutaneously into the posterior flanks of nude mice. Tumor volume was calculated using the formula (tumor volume  =  ½ length × width^2^) based on caliper measurements. Following tumor establishment, mice were randomly grouped and given 100 µl of Regorafenib (20 mg k^−1^g), SB525334 (20 mg k^−1^g), or AZ12601011 (20 mg k^−1^g) by oral gavage three times per week.

To established patient‐derived HCC tumor xenograft (PDX) models, tumor specimens were obtained from one HCC patient undergoing surgery in Daping hospital (Male, 53 years old), according to the protocols approved by the Hospital Institutional Review Board with informed consent from the participants. Another PDX was a precious gift from Dr. Cun Wang (State Key Laboratory of Systems Medicine for Cancer, Shanghai Cancer Institute, Renji Hospital, Shanghai Jiao Tong University School of Medicine), as descripted recently.^[^ ^47^ ^]^ The surgically resected tumor tissues were cut into fragments at approximately 2 × 2 × 2 mm^3^ and transplanted into NOD/SCID mice. Following tumor establishment, PDX mice were randomly grouped and treated three times per week with vehicle, Regorafenib (20 mg k^−1^g), or SB525334 (20 mg k^−1^g) or combination with Regorafenib and SB525334. The tumor volume was calculated as described above.

### Organoids

Patient‐derived organoids (PDOs) were cultured as previously described.^[^ ^48^ ^]^ Isolated patient‐derived tissue was minced and then incubated with digestion solution (125 µg ml^−1^ Dispase II, 80 U ml^−1^ Collagenase, 1X Pricomin in F12 medium) at 37 °C for 4–6 hours. Subsequently, the suspension was filtered through a 100 µm nylon cell strainer and centrifuged at 300 g for 5 minutes. The pellet was washed in cold Advanced DMEM/F12 (GIBCO) and mixed with Matrigel (Corning). Approximately 2000‐4000 cells were seeded per well in a 24‐well plate. After the Matrigel had solidified, the cells were cultured in a standard human liver organoid medium. This medium was composed of Advanced DMEM/F12 supplemented with 1% Penicillin/Streptomycin, 1% Glutamax, 10 mM HEPES, 1:50 B27 supplement, 1:100 N2 supplement, 1.25 mM n‐Acetyl‐L‐cysteine, 10 mM nicotinamide, 10 nM recombinant human Gastrin, 50 ng/ml recombinant human EGF, 200 ng ml^−1^ recombinant human FGF10, 25 ng ml^−1^ recombinant human HGF, 10 µM Y‐27632, 500 nM A83‐01, 10 µM SB202190, 1 µg ml^−1^ recombinant human R‐Spondin and 100 ng ml^−1^ recombinant Wnt‐3a(Wnt‐3a).

### Sphere Formation Assay and Limiting Dilution Assay

The procedures of spheroid formation was performed as previously described.^[^ ^46^ ^]^ Briefly, sphere‐initiating HCC cells were seeded into 24‐well ultra‐low adhesion plates (Corning, USA) and maintained in serum free F12/DMEM medium containing 20 ng mL^−1^ hEGF (PeproTech), 20 ng mL^−1^ bFGF (PeproTech) and 1X B27 supplement (Thermo). Cells were cultured in an incubator at 37 °C with 5% CO_2_ for 10 days and tumor spheres were then observed and photographed using a light microscope.

For in vitro limiting dilution assay, increasing cell numbers were cultured in sphere formation medium in 96‐well ultra‐low adhesion plates for 10 days. The numbers of wells that formed at least one tumor‐sphere were counted. For in vivo limiting dilution assay, a definite dose of indicated cells was injected subcutaneously into NOD/SCID mice. After three months, mice with xenografts in each group was counted. The extreme limiting dilution assays using ELDA website (http://bioinf.wehi.edu.au/software/elda/) was used for calculating the frequency of sphere‐initiating cells and cancer‐initiating cells.

### Colony Formation Assay and Soft Agar Colony Formation Assay

For colony formation assays, cells were seeded into 12‐well plates at a density of 500–2000 cells per well according to growth rate. After adherence, cells were treated with indicated drugs for 48 hours and and then cultured in normal DMEM for another 10–14 days. Cells were then fixed with 4% formaldehyde and stained with crystal violet diluted in water. Following two washes with PBS and air drying, images were captured using a digital camera and manually quantified.

Soft agar colony formation assays were performed to assess stemness of HCC cells under the anchorage‐independent conditions as previously described.^[^ ^49^ ^]^ In brief, 1 ml of DMEM containing 1% agar was added to 12‐well plates. After agar solidified, 1 ml DMEM containing 0.5% agar with 1 × 10^3^ HCC cells were seeded onto the gel layer. Subsequently, the cells were cultured for 3 to 4 weeks in an incubator at 37 °C with 5% CO_2_ and the colonies were stained with 1 mg ml^−1^ iodonitrotetrazolium chloride (Sigma, Germany).

### Western Blotting

Cells were washed by PBS and lysed in RIPA buffer containing protease inhibitor and phosphatase inhibitor cocktails (Bimake, USA). The protein concentration was measured and normalized using the BCA Protein Assay Kit (Beyotime, China), according to the manufacturer's instructions. Total protein was separated with SDS‐PAGE followed by transferring to polyvinylidene difluoride (PVDF) membranes (Millipore, USA). Membranes were subsequently blocked with 5% non‐fat milk diluted in TBS‐T for one hour. Then, the PVDF membranes were incubated with indicated primary antibody overnight at 4°C, followed by probing with HRP‐conjugated secondary antibody. Finally, chemiluminescence substrate (Share‐Bio, China) was added to the membranes and the blots was visualized with ChemiDoc imaging System (Bio‐Rad, USA). The detailed information of antibodies used in this study was described in Table S2 (Supporting Information).

### Immunohistochemical Staining and Scoring

For tissue microarrays containing HCC specimens, immunohistochemical staining was performed to detect the expression of TGFBRAP1, TGFΒR1 and p‐SMAD3 as previously described.^[^ ^49^ ^]^ In brief, the formalin‐fixed paraffin‐embedded samples underwent deparaffinization and rehydrated in graded ethanol. Antigens were retrieved by EDTA, and tissues were blocked by goat serum. Then sections were incubated with the indicated antibodies overnight at 4 °C. After incubation with horseradish peroxidase (HRP)‐conjugated secondary antibodies, DAB chromogenic solution was used to visualize the positive cells and hematoxylin staining to counterstain the cell nucleus. Final score of TGFBRAP1, TGFΒR1 and p‐SMAD3 in each sample was obtained by multiplying the distribution score and the strength score. A double‐blind method were employed to rigorously evaluate the IHC staining results, which were performed by two independent pathologists. The cutoff score in various analyses was 8 for staining intensity (high expression, IHC scoring ≥ 8; low expression, IHC scoring < 8).^[^ ^50^ ^]^

### Immunofluorescence Staining

For immunofluorescence analysis, cells were fixed in 4% paraformaldehyde for 20 min at room temperature. After washing three times with PBS, the fixed cells were permeabilized in 0.1% Triton X‐100 for 10 min and subsequently blocked in 10% bovine serum albumin (BSA) for 1 hour at room temperature. Primary antibody was diluted in PBS at the indicated dilution and incubated overnight at 4 °C. After washing three times with PBS+Tween‐20 (PBS‐T), the secondary antibody (Alexa Fluor 488, Invitrogen, dilution 1:500) was diluted in PBS‐T and incubated for 1 hour. Cells were washed with PBS and mounted with DAPI to counterstain the nucleus. The fluorescence image was taken by Leica microscope (DMi8), and analyzed by ImageJ software.

### RNA Extraction and qRT‐PCR

Total RNA was extracted with Trizol reagent (Invitrogen) and reverse‐transcribed using the PrimeScript RT Reagent Kit (Takara) to synthesize cDNA according to the manufacturer's instructions. SYBR Premix Ex Taq (Takara) was used to amplify cDNA and detect the Ct value of indicated genes with a CFX96 Real Time PCR Detection System (Bio‐Rad). Primers used in these studies were listed in Table S3 (Supporting Information).

### Luciferase

Luciferase reporter assays were performed as previously described.^[^ ^51^ ^]^ Briefly, cells were seeded in 24‐well plates and transfected with indicated plasmids with lipofectamine3000 according to the manufacturer's instruction. Approximately 100 ng of CAGA luciferase or *TGFBRAP1*‐promoter luciferase reporter plasmids and 10 ng of renilla luciferase reporter plasmid were co‐transfected. Cells were harvested 48 hours after transfection and the luciferase activity was measured with the Dual Luciferase Reporter Assay System (Promega). Relative luciferase activity was calculated as firefly luminescence relative to renilla luminescence.

### Co‐Immunoprecipitation Assay

For the co‐immunoprecipitation assays, HEK293T cells were co‐transfected with indicated plasmids for 48 hours and lysed with immunoprecipitation lysis buffer (Thermo Fisher Scientific, USA) or HCC cells were used to measure endogenous binding of different proteins. Cell lysates were incubated with indicated antibody and Protein A/G beads overnight at 4 °C. The precipitated protein complexes were washed with lysis buffer three times and eluted with 2X SDS loading buffer. The immunoprecipitates were separated by SDS‐PAGE for western blotting analysis.

### Protein Half‐Life Assays

To detect the half‐life of TGFBR1 protein in HCC cells, cycloheximide (CHX) assays were performed as were previously described.^[^ ^51^ ^]^ Briefly, 20 µM CHX was added to the cell medium and total protein was collection at indicated time points. Protein levels were measured via western blotting.

### Chromatin Immunoprecipitation (ChIP)

The ChIP assay was conducted utilizing the EZ ChIP Kit following the manufacturer's protocol. In brief, cells were cross‐linked with 1% formaldehyde and resuspended in lysis buffer. Then cells were sonicated to generate DNA fragments and centrifuged to obtain supernatants. Then the supernatants were incubated with indicated primary antibodies and Protein G beads overnight at 4 °C. The ChIP products were de‐crosslinked with 200 mM NaCl at 65 °C overnight and analyzed by qRT‐PCR. The primers used for ChIP analysis were also listed in Table S3 (Supporting Information).

### Ubiquitination Assays

Cellular ubiquitination assays were performed as were previously described.^[^ ^49^ ^]^ Briefly, HEK293 cells were transiently transfected with indicated plasmid with or without His‐Ub for 48 hours and 10 µM MG132 was added into culture media 12 hours before collection. Cells were lysed in denaturing Buffer A (6 M guanidine‐HCl, 0.1 M Na_2_HPO_4_/NaH_2_PO_4_, 10 mM imidazole, pH 8.0) and sonication was performed to fully lyse the cells. Ni‐NTA resin (QIAGEN) was used to enrich for poly‐ubiquitinated proteins for 5 hours at room temperature. Subsequently, the pull‐down precipitation products were washed once with Buffer A, twice with Buffer A/TI (1:3) mixture solution, and finally once with Buffer TI (25 mM Tris‐HCl and 20 mM imidazole, pH 6.8). The pull‐down products were boiled in 50 µl 2X Laemmli SDS loading buffer and were separated by SDS‐PAGE for western blotting analysis.

### Statistical Analysis

GraphPad Prism version 8 was used for statistical analysis, and the majority of experiments were repeated at least three times to obtain representative data. All statistical data were presented as mean±SEM and means between two groups were analyzed by two‐tailed Student's t‐test and multiple comparisons were performed with one‐way ANOVA or two‐way ANOVA. For all statistical tests, a value of *p*<0.05 was considered statistically significant.

### Ethics Approval

Army Medical University Medical Ethics Committee (Number, SYXK 20170002).

## Conflict of Interest

The authors declare no conflict of interest.

## Author Contributions

K.L., F.T., X.C., and B.L. authors share co‐first authorship. K.L. wrote the manuscript. K.L., F.T., X.C., B.L., Z.C., J.L. performed experiments. S.T., Y.H., L.W., M.H., S.P., Y.T., Y.P., X.L., and Z.Q. analyzed data. L.C., D.C., L.W., J.X., and X.‐W.B. provided technical support. Q.L., X.Y., T.W., and B.W. designed the research, interpreted data, and supervised the study. B.W. edited the manuscript and approved the submission.

## Supporting information

Supporting Information

## Data Availability

The data that support the findings of this study are available from the corresponding author upon reasonable request.
