# Supplementary material for: Stabilization of TGF‐β Receptor 1 by a Receptor‐Associated Adaptor Dictates Feedback Activation of the TGF‐β Signaling Pathway to Maintain Liver Cancer Stemness and Drug Resistance
Source: Adv Sci (Weinh). 2024 Jul 9;11(34):2402327. doi: 10.1002/advs.202402327 (PMC11425868; doi:10.1002/advs.202402327)
Supplement: Supplementary file 1 — Supporting Information [file ADVS-11-2402327-s001.pdf]

ADVANCED SCIENCE
Open Access

Supporting Information

for *Adv. Sci.*, DOI 10.1002/advs.202402327

Stabilization of TGF-β Receptor 1 by a Receptor-Associated Adaptor Dictates Feedback Activation of the TGF-β Signaling Pathway to Maintain Liver Cancer Stemness and Drug Resistance

Kewei Liu, Fanxuan Tian, Xu Chen, Biyin Liu, Shuoran Tian, Yongying Hou, Lei Wang, Mengyi Han, Shiying Peng, Yuting Tan, Yuwei Pan, Zhaole Chu, Jinyang Li, Linrong Che, Dongfeng Chen, Liangzhi Wen, Zhongyi Qin, Xianfeng Li, Junyu Xiang, Xiu-wu Bian, Qin Liu, Xiaoli Ye*, Tao Wang* and Bin Wang**

Supporting Information for

Stabilization of TGF-β Receptor 1 by a Receptor-associated Adaptor Dictates Feedback Activation of the TGF-β Signaling Pathway to Maintain Liver Cancer Stemness and Drug Resistance

Figure S1-6 and figure legends

Table S1-3

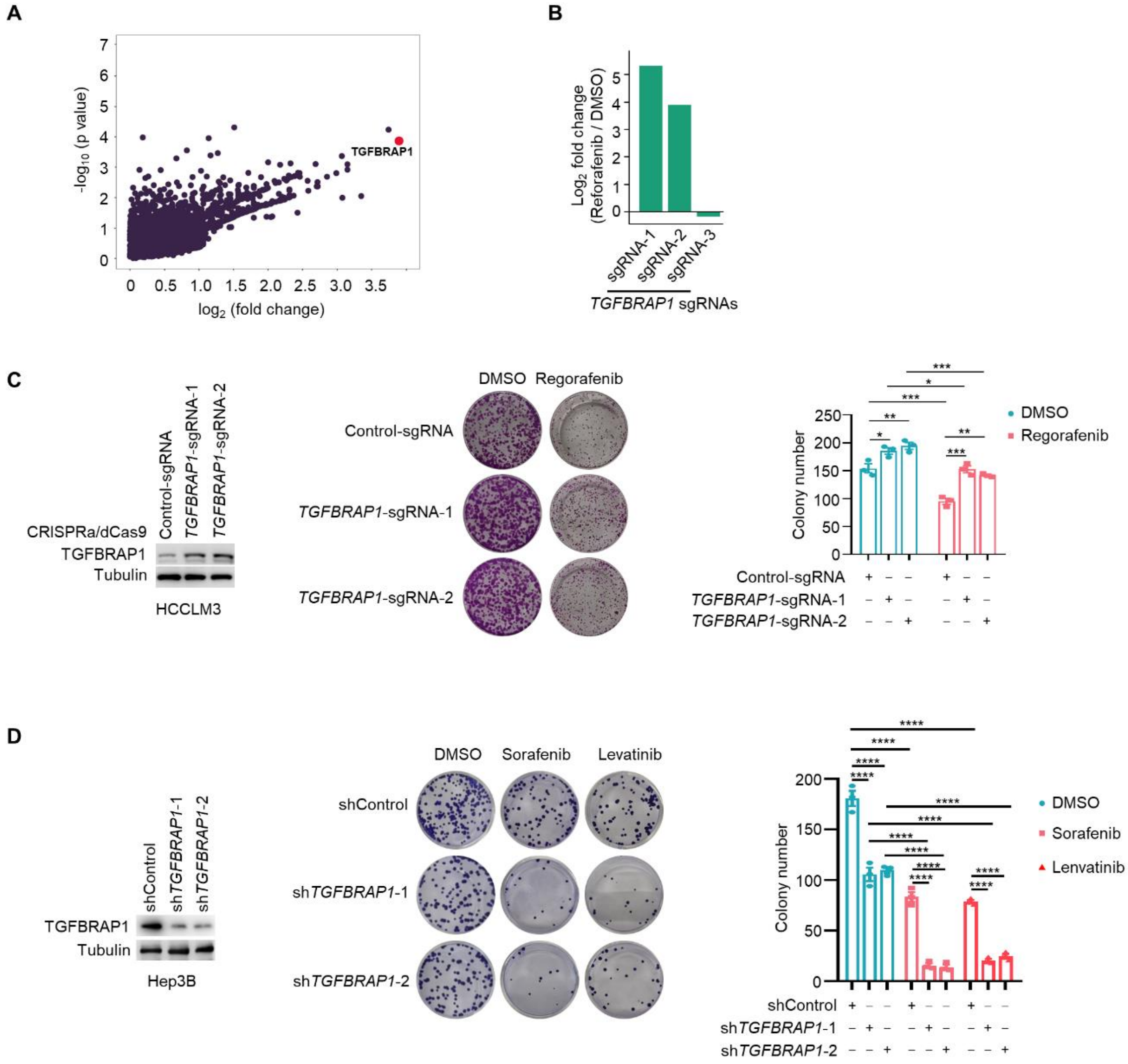

Figure S1. TGFBRAP1 promotes resistance of HCC cells to multiple tyrosine kinase inhibitors.
(A) Scatter plot of sequencing results from screen according to the enrichment score based on the Log2 (fold change) and *p*-value. (B) Changes in each sgRNA targeting *TGFBRAP1* represented in the CRISPR activation screen. (C) Western blotting analysis of cell lysates of HCCLM3 cells infected lentivirus carrying a CRISPRa-dCas9 activation system and control or *TGFBRAP1*-targeting sgRNA to induce moderate overexpression of TGFBRAP1. Representative images (middle) and quantitative results (right) of colony formation assays were shown. The cells were treated with DMSO or Regorafenib (10 μM) to evaluate drug sensitivity. (D) Western blotting analysis to validate the knockdown efficacy of shRNA targeting *TGFBRAP1* in Hep3B cells (left). Representative images (middle) and quantitative results (right) of colony formation assays were shown, with cells treated with either DMSO or Sorafenib (10 μM), or Levatinib (10 μM). *p <0.05, **p <0.01 , ***p <0.001 and ****p <0.0001.

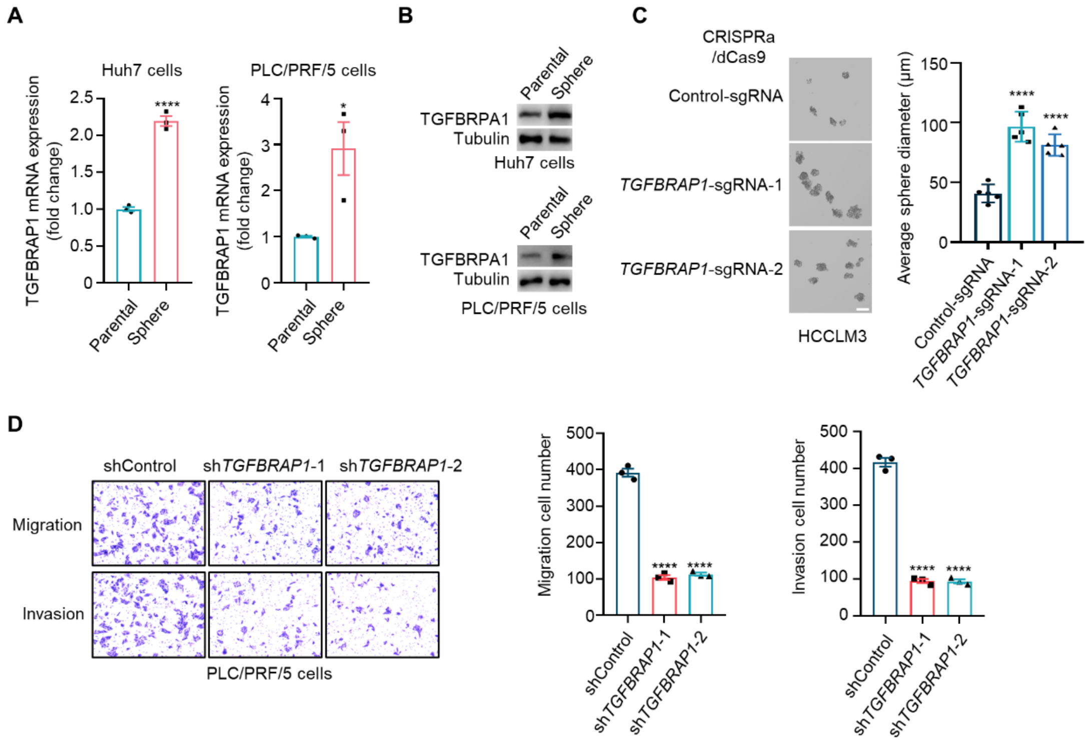

E

Huh7 cells CRISPRa/dCas9	Number of tumors/total injections cells per injection 1x10^6	1x10^5	1x10^4	1x10^3	Tumor-initiating frequency Estimate (95% confidence interval)			Fold	p-Value
Control-sgRNA	6/6	5/6	3/6	2/6	1/27214	1/71233	1/10397	1	
TGFBRAP1-sgRNA-1	6/6	6/6	5/6	4/6	1/3006	1/7863	1/1150	9.05	< 0.001
TGFBRAP1-sgRNA-2	6/6	6/6	5/6	3/6	1/9169	1/3655	1/1457	7.44	< 0.001

Figure S2. TGFBRAP1 is elevated in cancer stem cell-like cells to maintain stemness properties.
(A) TGFBRAP1 mRNA expression levels in Huh7 and PLC/PRF/5 cells under tumor sphere culture condition or normal condition (parental). (B) Western blotting analysis of TGFBRAP1 protein levels in the lysates of Huh7 and PLC/PRF/5 cells under either parental or sphere culture conditions. (C) Representative images of spheres formed by control or *TGFBRAP1*-overexpressing HCCLM3 cells and quantification of sphere diameter. Scale bar represents 100 μm. (D) The migration and invasion assays using control or *TGFBRAP1*-knockdown PLC/PRF5 cells. (E) Frequencies of cancer-initiating cells (CICs) of Huh7 cells infected lentivirus carrying a CRISPRa-dCas9 activation system and control or *TGFBRAP1*-targeting sgRNA to induce moderate overexpression of TGFBRAP1, as analyzed by extreme limiting dilution assays in NOD/SCID mice. *p <0.05 and ****p <0.0001.

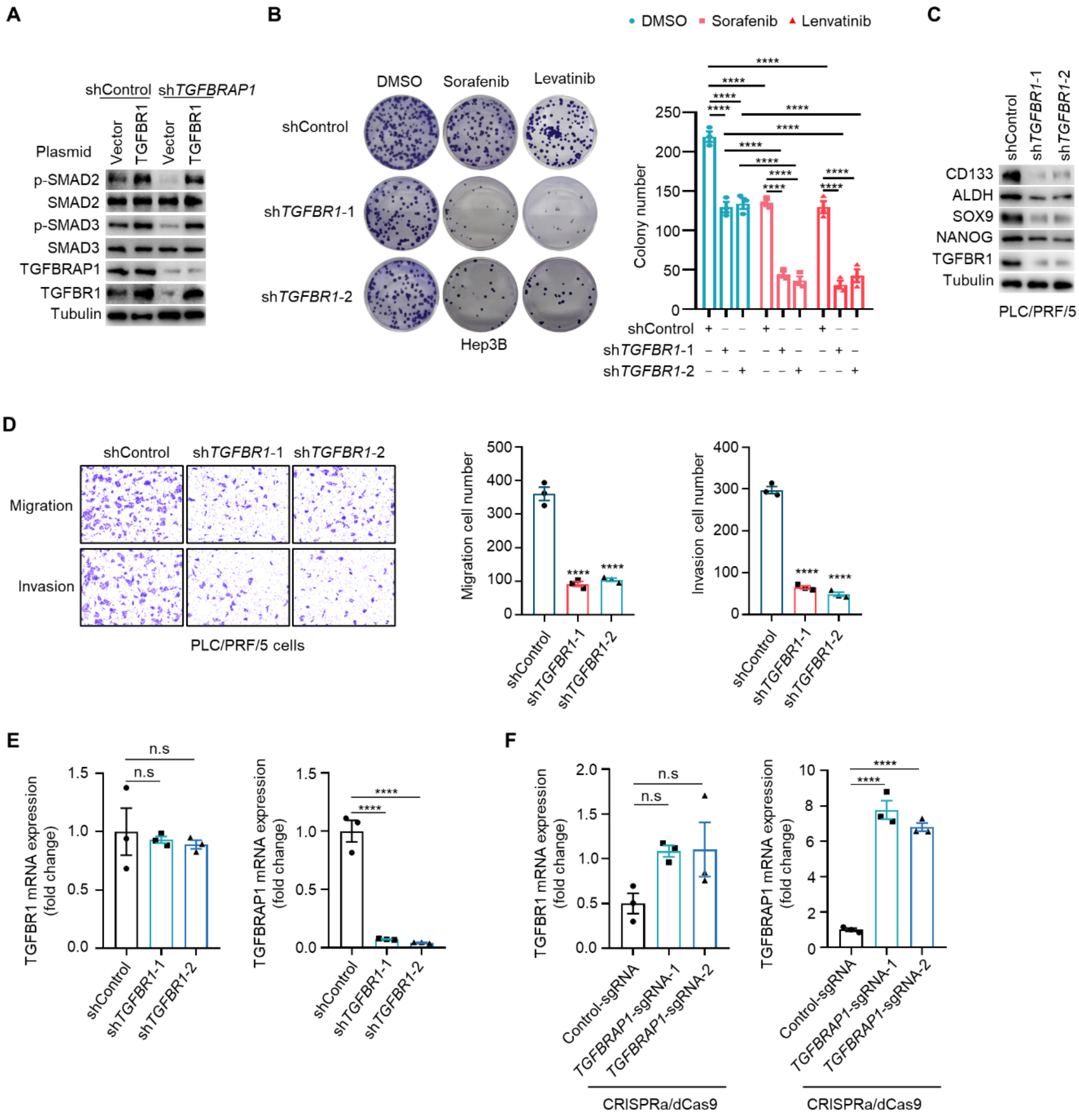

Figure S3. TGFBRAP1 potentiates TGF-β signaling pathway by up-regulating TGFBR1 expression. (A) Western blotting analysis of p-SMAD2 and p-SMAD3 levels in indicated cells. (B) Representative images of colony formation assays, quantification of colony number in control or *TGFBR1*-knockdown Hep3B cells, as treated with either DMSO, or Sorafenib (10 μM), or levatinib (10 μM)). (C) Western blotting analysis of CD133, ALDH, SOX9, and NANOG in control or *TGFBR1*-knockdown cells. (D) The migration and invasion assays using control or *TGFBR1*-knockdown cells. (E) mRNA expression levels of *TGFBR1* and *TGFBRAP1* in *TGFBRAP1*-depleted cells. (F) mRNA expression levels of TGFBR1 and TGFBRAP1 in *TGFBRAP1*-overexpressing cells. Statistical analyses were performed by one-way ANOVA comparisons test. ****p <0.0001.

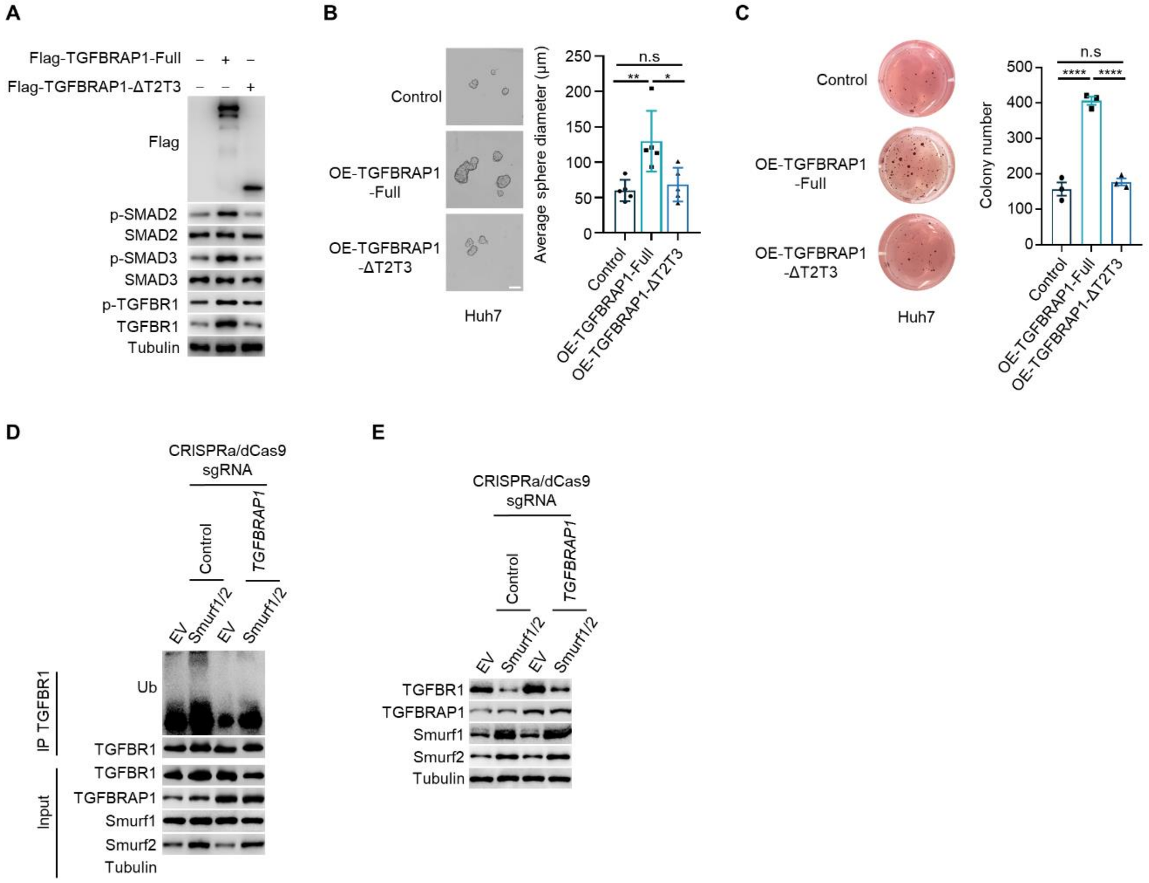

Figure S4. TGFBRAP1 interacts with TGFBR1 to prevent its degradation, thereby maintaining cancer stemness properties.

(A) Western blotting analysis of p-SMAD2, p-SMAD3 and p-TGFBR1 levels in Huh7 cells overexpressing full-length TGFBRAP1 or TGFBR1-non-interacting TGFBRAP1 mutant that was deleted with both the T2 and T3 domains (ΔT2T3). (B) Representative images of spheres formed by control Huh7 cells, or cells expressing the full length TGFBRAP1 or TGFBR1-non-interacting TGFBRAP1 mutant (left). The sphere diameter were quantified on the right. Scale bar represents 100 μm. (C) Representative images of soft agar colony formation assays and quantification of colony number in indicated cells. (D) Western blotting analysis of whole cell lysates and immunoprecipitation to detect ubiquitination of TGFBR1 in indicated Huh7 cells treated with MG132. (E) Western blotting analysis of TGFBR1 and TGFBRAP1 levels in indicated Huh7 cells. *p <0.05, **p <0.01 and ****p <0.0001.

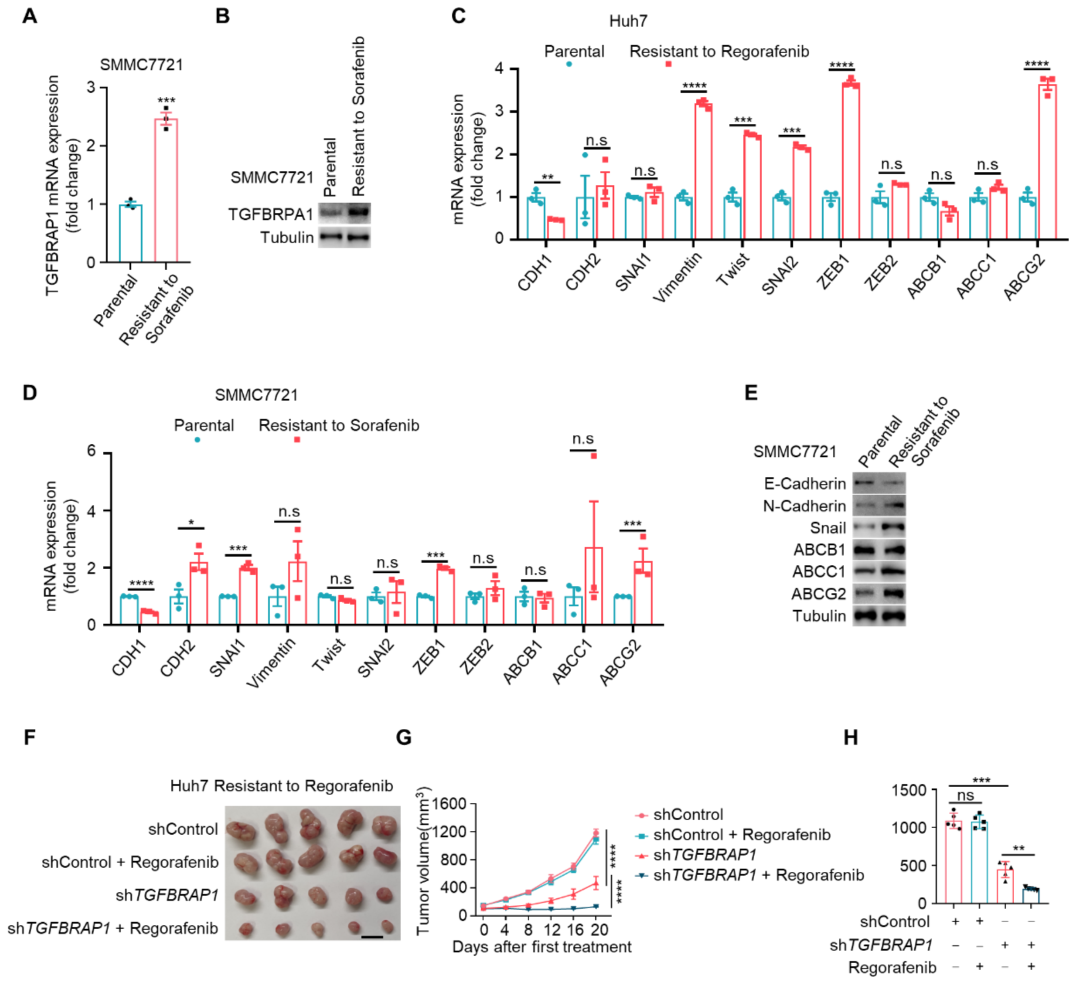

Figure S5. TGFBRAP1 is up-regulated in TKI-resistance cells to confer drug insensitivity, while it moderately affects the EMT process.

(A)TGFBRAP1 mRNA expression in the parental or Sorafenib-resistant SMMC7721 cells. (B) TGFBRAP1 protein levels in the parental or Sorafenib-resistant SMMC7721 cells. (C) mRNA expression of EMT and multidrug resistance markers in the parental or Regorafenib-resistant Huh7 cells. (D) mRNA expression of EMT and multidrug resistance markers in the parental or Sorafenib-resistant SMMC7721 cells. (E) Western blotting analysis of indicated EMT and multidrug resistance markers in the parental or Sorafenib-resistant SMMC7721 cells. (F) Representative xenograft tumors generated by control or *TGFBRAP1*-depleted Regorafenib-resistant Huh7 cells in nude mice, which were treated with vehicle or Regorafenib (20 mg/kg). (G) Growth curves of xenografts. (H) The quantification of tumor weights in each group. *p <0.05, **p <0.01 , ***p <0.001 and ****p <0.0001.

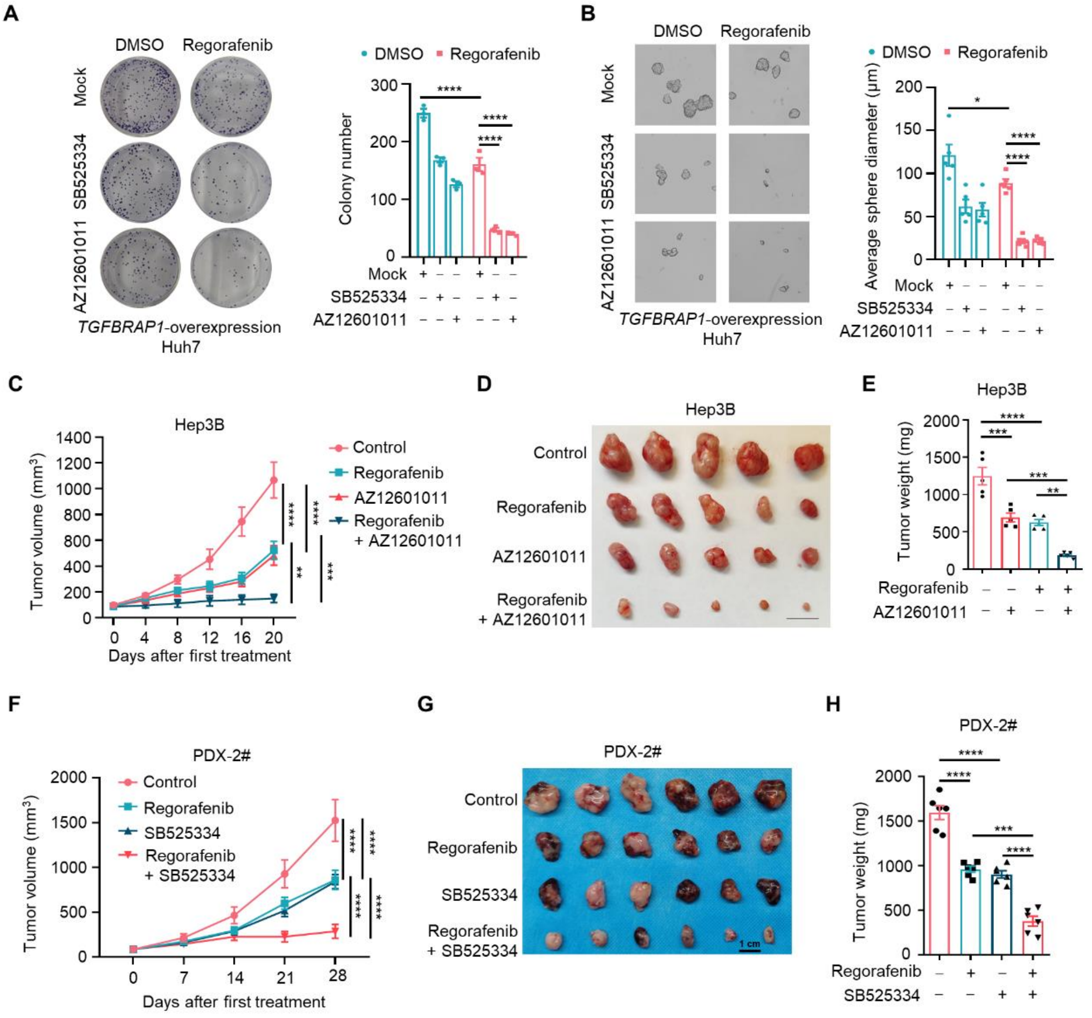

Figure S6. Inhibition of TGFBR1 in *TGFBRAP1*-overexpressing cells overcomes resistance to the TKI Regorafenib.

(A) Colony formation assays of Huh7 cells-overexpressing endogenous *TGFBRAP1* as generated in **Figure 1C**, in the presence of Regorafenib (10 μM), SB525334 (10 μM) or AZ12601011 (10 μM) alone, or in combination. (B) Spheres formed by *TGFBRAP1*-overexpressing Huh7 cells treated as indicated compounds. Scale bar represents 100 μm. (C) Growth curves of xenografts derived from Hep3B cells grown in nude mice treated with either Regorafenib (20 mg/kg)or AZ12601011(20 mg/kg), alone or in combination. Scale bar represents 1 cm. (D) Representative xenografts at endpoint. (E) Quantification of tumor weights in each group at endpoint. (F) Growth curves of HCC PDX-2# in NOD/SCID mice treated with either Regorafenib (20 mg/kg) or SB525334 (20 mg/kg) alone or in combination. Scale bar represents 1 cm. (G) Representative PDX tumors at endpoint. (H) The PDX tumor weights in each group. Statistical analyses were performed by two-way ANOVA with Bonferroni's multiple comparisons test. *p <0.05, **p <0.01 , ***p <0.001 and ****p <0.0001.

Table S1. Correlation between TGFBRAP1 protein levels in HCC tissues and related clinicopathologic parameters

Parameters	Cases	TGFBRAP1 expression	*p*-value

	191	Low 86 (%)	High 105 (%)	
**Gender**				
male	154	72 (46.8%)	82 (53.2%)	0.3277
female	37	14 (37.8%)	23 (62.2%)	
**Age (years)**				
< 60	139	65 (46.8%)	74 (53.2%)	0.4303
≥60	52	21 (40.4%)	31 (49.6%)	
**Tumor size (Diameter, cm)**				0.0129*
< 5	56	33 (40.4%)	23 (59.6%)	
≥5	135	53 (39.3%)	82 (60.7%)	
**Histological Grade**				
high (Ⅰ+Ⅱ)	67	40 (59.7%)	27 (40.3%)	0.0027**
low (III)	124	46 (37.1%)	78 (62.9%)	
**Number of tumor(s)**				
single	133	63 (40.7%)	70 (52.6%)	0.3245
multiple	58	23 (39.7%)	35 (60.3%)	
**Tumor capsule**				
complete	99	46 (46.5%)	53 (53.5%)	0.6785
none	92	40 (43.5%)	52 (46.5%)	
**Liver cirrhosis**				
no	79	35 (44.3%)	44 (55.7%)	0.8662
yes	112	51 (45.5%)	61 (54.5%)	
**Lymphatic invasion**				
no (N0)	109	53 (48.6%)	56 (51.4%)	0.2492
yes (N1)	82	33 (40.2%)	49 (59.8%)	
**TNM Stage**				
Ⅰ+Ⅱ	66	41 (62.1%)	25 (37.9%)	0.0006***
III+Ⅳ	125	45 (36.0%)	80 (64.0%)	
**Vessel invasion**				
no	171	77 (45.0%)	94 (55.0%)	0.9980
yes	20	9 (45.0%)	11 (55.0%)	

Table S2. Antibodies used in this study.

**Antibody**	**Vender**	**Cat. #**	**Dilution**
TGFBRAP1	Proteintech	20153-1-AP	1:1000 (WB)
TGFBRAP1	Sigma	HPA038397	1:100 (IP)<br>1:200 (IHC)

Tubulin	Proteintech	HRP-66031	1:2000 (WB)
SMAD2	CST	#5339	1:1000 (WB)
p-SMAD2	Abclonal	AP0269	1:1000 (WB)
SMAD3	CST	#5923	1:1000 (WB)
p-SMAD3	Abclonal	AP0727	1:1000 (WB)<br>1:200 (IHC, ICC)
SMUFR1	CST	#2174S	1:1000 (WB)
SMURF2	Abclonal	A10592	1:1000 (WB)
TGFBR1	Abclonal	A0708	1:1000 (WB)
TGFBR1	Abcam	ab235578	1:200(IHC)<br>1:100(IP)
p-TGFBR1	Affinity	AF8456	1:1000 (WB)
TGFBR2	Bioss	bs-0117R	1:1000 (WB)
p-TGFBR2	Affinity	AF8191	1:1000 (WB)
OCT4	Abclonal	A7920	1:1000 (WB)
NANOG	Proteintech	14295-1-AP	1:1000 (WB)
SOX9	Abclonal	A19710	1:1000 (WB)
Ubiquitin	Abways	CY5520	1:1000 (WB)
E-cadherin	Proteintech	60335-1-Ig	1:1000 (WB)
N-Cadherin	Beyotime	AF5237	1:1000 (WB)
Snail	CST	#14074	1:1000 (WB)
ABCB1	Abclonal	A11758	1:1000 (WB)
ABCC1	Abclonal	A11153	1:1000 (WB)
ABCG2	Abclonal	A17908	1:1000 (WB)
Flag-tag	MBL	M185-3	1:1000 (WB)<br>1:200 (IP)
Myc-tag	MBL	M192-3	1:1000 (WB)<br>1:200 (IP)
HA-tag	MBL	M180-3	1:1000 (WB)<br>1:200 (IP)

CST: Cell Signaling Technology

Table S3. Primers used in this study.

Gene	Application	Sense (5'-3')	Antisense(5'-3')
Tgfbrap1	qRT-PCR	CTGCCTGGCTAGAGAAGCACAA	TCATACAGGTCTGAGCGTGTGG
Tgfbrap1	sgRNA for dCas9-activation	1#: GTGGCCCCGGTGCACGGAGG<br>2#: ACACGGCCCGCCTCGCGGGG	1#: CCTCCGTGCACCGGGGCCAC<br>2#: CCCCGCGAGGCGGGCCGTGT
Tgfbrap1	shRNA	1#: GCTCAGACCTGTATGAATACA<br>2#: GGTAATGTACAGAAGGATTCT	1#: TGTATTCATACAGGTCTGAGC<br>2#: AGAATCCTTCTGTACATTACC

β-actin	qRT-PCR	CACCATTGGCAATGAGCGGTTC	AGGTCTTTGCGGATGTCCACGT
Smad2	qRT-PCR	GGGTTTTGAAGCCGTCTATCAGC	CCAACCACTGTAGAGGTCCATTC
Smad3	qRT-PCR	TGAGGCTGTCTACCAGTTGACC	GTGAGGACCTTGTCAAGCCACT
Tgfbr1	qRT-PCR	GACAACGTCAGGTTCTGGCTCA	CCGCCACTTTCCTCTCCAAACT
CDH1	qRT-PCR	GCCTCCTGAAAAGAGAGTGGAA	TGGCAGTGTCTCTCCAAATCCG
CDH2	qRT-PCR	CCTCCAGAGTTTACTGCCATGAC	GTAGGATCTCCGCCACTGATTC
SNAI1	qRT-PCR	TGCCCTCAAGATGCACATCCGA	GGGACAGGAGAAGGGCTTCTC
Vimentin	qRT-PCR	AGGCAAAGCAGGAGTCCACTGA	ATCTGGCGTTCCAGGGACTCAT
Twist	qRT-PCR	GCCAGGTACATCGACTTCCTCT	TCCATCCTCCAGACCGAGAAGG
SNAI2	qRT-PCR	ATCTGCGGCAAGGCGTTTTCCA	GAGCCCTCAGATTTGACCTGTC
ZEB1	qRT-PCR	GGCATACACCTACTCAACTACGG	TGGGCGGTGTAGAATCAGAGTC
ZEB2	qRT-PCR	AATGCACAGAGTGTGGCAAGGC	CTGCTGATGTGCGAACTGTAGG
ABCB1	qRT-PCR	GCTGTCAAGGAAGCCAATGCCT	TGCAATGGCGATCCTCTGCTTC
ABCC1	qRT-PCR	CCGTGTACTCCAACGCTGACAT	ATGCTGTGCGTGACCAAGATCC
ABCG2	qRT-PCR	GTTCTCAGCAGCTCTTCGGCTT	TCCTCCAGACACACCACGGATA
Smurf1	sgRNA	AACCCCGGGACACGCAGGAA	TTCCTGCGTGTCCCGGGGTT
Smurf2	sgRNA	GCTTGACGGGCCCGTTCCTC	GAGGAACGGGCCCGTCAAGC
Smad2	siRNA	GGTCTTGCCTCTAAACTATAT	ATATAGTTTAGAGGCAAGACC
Smad3	siRNA	GCACTGACCATAAGAGCAACA	TGTTGCTCTTATGGTCAGTGC
Tgfbrap1	ChIP-qPCR	TAGCGTCTACTTCATTGCTC	GCTCACAGAGACTATGTGAC
